# Bacterial Cellulose/Polypyrrole Aerogel for the Efficient Removal of Organic and Inorganic Water Contaminants

**DOI:** 10.1021/acsomega.6c00432

**Published:** 2026-04-08

**Authors:** Islam M. Minisy, Radim Striz, Zuzana Morávková, Adriana Kovalcik, Patrycja Bober

**Affiliations:** † Institute of Macromolecular Chemistry, 86879 Czech Academy of Sciences, 162 00 Prague, Czech Republic; ‡ Faculty of Chemistry, 649431 Brno University of Technology, Purkynova 118, 612 00 Brno, Czech Republic

## Abstract

In this work, in situ oxidative polymerization of pyrrole in the presence of bacterial cellulose (BC) hydrogels was utilized to prepare BC/polypyrrole (PPy) aerogels. Various analytical techniques, such as scanning electron microscopy with energy-dispersive X-ray spectroscopy, thermal gravimetric analysis, Fourier transform infrared (FTIR) and Raman spectroscopies, and BET specific surface area analysis, were employed to characterize the BC/PPy composite. Raman and FTIR spectroscopy confirmed the formation of doped PPy that uniformly coated the BC fibers. BC/PPy has demonstrated excellent adsorption capability of organic (Reactive Black 5, RB) and inorganic (hexavalent chromium ions, Cr­(VI)) wastewater contaminants in single and binary solute systems. Various parameters, e.g., contact time, pH, and adsorbent dosage, were evaluated in batch sorption experiments. The adsorption processes were found to obey pseudo-second-order kinetics and Langmuir isotherm models. Vibrational spectroscopy indicates that the Cr­(VI) adsorption involves redox reactions, while RB adsorption occurs through ion exchange. The maximum adsorption capacity of 485.4 and 130.7 mg g^–1^ of Cr­(VI) and RB was achieved at pH 2 and 4, respectively. After five adsorption/desorption cycles, BC/PPy maintained removal efficiencies of 92.1 and 97.7% for Cr­(VI) and RB, respectively. This research demonstrates the cost-effectiveness, reusability, and sustainability of BC/PPy as an adsorbent for removing pollutants from wastewater.

## Graphical abstract

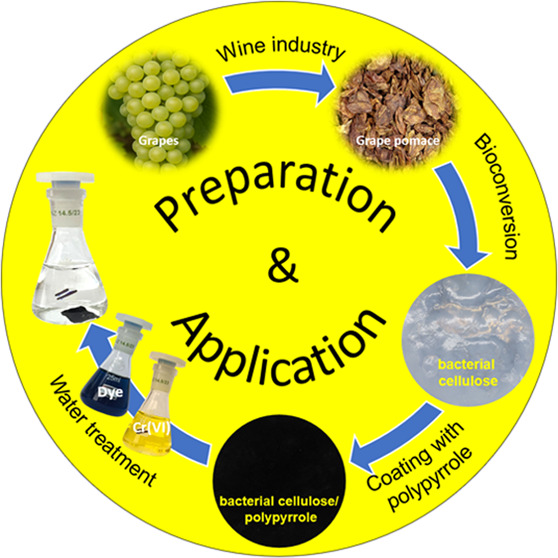

## 1 Introduction

Heavy metals, e.g., Hg, Cd, Pb, Cr, etc., can pose various health and environmental risks, especially when dissolving in water bodies.^1^ Organic dyes are no less dangerous than heavy metals.^2^ Their accumulation can lead to lethal complications for human beings, animals, aquatic life, and ecosystems. Among these pollutants, hexavalent chromium ions (Cr­(VI)) and Reactive Black 5 (RB) organic dye are commonly found in industrial wastewater. Cr­(VI), primarily released from industrial activities, is highly toxic, carcinogenic, and can cause serious damage to the liver, kidneys, and respiratory system even at low concentrations.^3^ RB, a synthetic azo dye widely used in textile industries, is similarly resistant to biodegradation, and its toxicity increases after hydrolysis.^4,5^ Due to the high urbanization activities, large amounts of wastewater contaminated with heavy metals (Cr­(VI)) and organic dyes (RB) leak into the clean water sources.^6^ This requires effective water treatment solutions to promote sustainable water management and ensure a reliable and clean water supply. Various methodologies are employed to remediate wastewater contaminated with heavy metals and organic dyes, such as membrane separation, flocculation, coagulation, sedimentation, precipitation, and advanced oxidation technologies.^7^ Due to high operational costs and environmental impacts, these approaches might encounter feasibility limitations on industrial scales. Adsorption stands out among these approaches for its inherent benefits, including effective removal with the ability to target trace levels of pollutants, simplicity in operation and handling, minimal sludge generation, ease of adsorbent regeneration, and cost-effectiveness.^8–10^ The discovery of highly efficient adsorbents is essential for advancing adsorption technology. Moreover, from a sustainability perspective, the development of environmentally friendly and biobased materials for removing water contaminants is of great importance.

Compared to conventional cellulose, bacterial cellulose (BC) has shown attractive properties such as higher purity, crystallinity, mechanical strength, water-holding capacity, and biocompatibility.^11,12^ BC consists of 3D interconnected network of pure cellulose fibers synthesized by bacteria.^13^ Compared to plant cellulose, it has no lignin and hemicellulose, which are typically removed under harsh conditions using toxic chemicals.^14^ In addition, BC has a high surface area and antifouling properties that prevent contaminants from building up on its surface. These properties make BC a preferred choice over conventional cellulose for preparing functional aerogels for wastewater treatment applications, ensuring effective removal of pollutants and long-term stability.^15^ However, low-yield and time-consuming processes are still challenging for large-scale production.^16^ Recent studies have demonstrated the efficiency of BC in the adsorptive removal of various water contaminants, including heavy metals and organic dyes.^17^ For instance, BC aerogel derived from pineapple peel waste exhibited limited adsorption capacities for most cationic dyes, including rhodamine B (28 mg g^–1^), methylene blue (29.7 mg g^–1^), crystal violet (33 mg g^–1^), and malachite green (48.6 mg g^–1^). Additionally, this aerogel showed no affinity for most anionic dyes, except Congo red, with an adsorption capacity of 101 mg g^–1^ driven by strong hydrogen bonds between its amine groups and the BC hydroxyls.^14^ To further enhance the adsorption capacity of BC, it was functionalized with conducting polymers such as polyaniline or polypyrrole (PPy).^18–20^ PPy is one of the most extensively studied conducting polymers for removing water contaminants due to its nontoxicity, cost-effectiveness, ease of synthesis, water insolubility, and high stability.^21,22^ Nevertheless, PPy also exhibits critical limitations, including low intrinsic adsorption capacity, low surface area, and poor processability, which restrict its overall efficiency. The preparation of PPy composites is the most effective strategy to produce more effective adsorbents with enhanced functionality and stability.^23^

Incorporating PPy into BC has enhanced the adsorption capacity of cationic dyes and heavy metals through electrostatic interactions and chelation.^14,19,24^ PPy/BC efficiently removed Cr­(VI) through chemisorption involving ion exchange, electrostatic interaction, and Cr­(VI) reduction to Cr­(III), with a maximum adsorption capacity of 555.6 mg g^–1^ at 25 °C and pH 2.^19^ It was found to maintain 70.5% of its initial adsorption capacity after five adsorption/desorption cycles.^19^ Recently, our group has developed a free-standing and easily separable PPy/BC composite^25^ with a specific surface area of 61.96 m^2^ g^–1^, which has been used for the adsorptive and photocatalytic removal of Cr­(VI) ions, demonstrating a maximum adsorption capacity of 294.1 mg g^–1^.^25^

In this work, we hypothesize that the use of BC cultivated on grape pomace as a carbohydrate source can contribute to reducing the waste and promote a circular economy, and the coating of BC with PPy composite can yield a sustainable and efficient adsorbent for the adsorptive removal of heavy metals and organic dyes. An in situ oxidative polymerization of pyrrole onto BC was performed to prepare BC/PPy composite, which was subsequently transformed into aerogels by freeze-drying. BC/PPy substrate was examined for removing Cr­(VI) ions and RB from aqueous solutions in single and binary mixture systems. To the best of our knowledge, BC/PPy composites have not been previously reported for the removal of organic dyes from wastewater. The single contaminant adsorption system was modeled using various kinetics and equilibrium isotherm models. The adsorption mechanism was investigated using FTIR and Raman spectroscopies, as well as scanning electron microscopy with energy-dispersive X-ray spectroscopy (SEM-EDX).

## 2 Experimental Section

### 2.1 Chemicals and Reagents

Peptone, yeast extract, and *N,N*-dimethylacetamide were obtained from HiMedia (India). Citric acid, magnesium sulfate heptahydrate, disodium phosphate dodecahydrate, sodium hydroxide, and lithium chloride were purchased from Lach-Ner (Czech Republic). Pyrrole (reagent grade, 98%), iron­(III) chloride hexahydrate (≥99%), potassium dichromate (K_2_Cr_2_O_7_, ≥99.5%), Reactive Black 5 (Remazol Black B, C_26_H_21_N_5_Na_4_O_19_S, *M*_w_ = 991.82 g mol^–1^), and Viscozyme L were purchased from Sigma-Aldrich (Germany). All reagents were used as received.

### 2.2 Biosynthesis of Bacterial Cellulose

BC hydrogels (Figure S1) were prepared by static cultivation of *Komagataeibacter xylinus* (ATCC 53524; Manassas, VA, USA) in a modified Hestrin-Schramm (MHS) medium, where an enzymatic hydrolysate was used as a base for other medium components. This hydrolysate was prepared following a previously published method with slight modifications.^26^ It was derived from seedless grape pomace originating from Grüner Veltliner and Sauvignon Blanc grapes (obtained from Vavricek winery, Czech Republic). The MHS medium contained 5 g L^–1^ peptone and yeast extract, 1.15 g L^–1^ citric acid, 6.8 g L^–1^ disodium phosphate dodecahydrate, 0.06 g L^–1^ magnesium sulfate, and diluted hydrolysate (20 g L^–1^ of sugars). After sterilization, the MHS medium was enriched with 1% (v/v) ethanol and inoculated with 10% of a 7-day-old bacterial suspension of 2 × 10^5^ CFU ml^–1^. BC production was performed in 90 mm Petri dishes with 25 mL of MHS for 14 days under static conditions at 30 °C. Afterward, BC pellicles were removed and purified with 0.1 M NaOH at 80 °C for 90 min and rinsed with distilled water until a neutral pH was reached. BC hydrogels were stored in 70% ethanol at 4 °C for further use.

### 2.3 Preparation of Bacterial Cellulose/Polypyrrole Composite

The BC/PPy composite was prepared through in situ chemical oxidative polymerization of pyrrole in the presence of BC hydrogels at room temperature.^25^ First, BC hydrogels were pressed between two Petri dishes to expel excess absorbed water, then soaked in 0.2 M pyrrole solution (200 mL) for 1 h with stirring. The solution containing BC was then kept in the refrigerator overnight. Finally, it was mixed with 200 mL of iron­(III) chloride solution (0.5 M), stirred for a few minutes, and allowed to stand for 1 h. The final BC/PPy hydrogels were washed with a large volume of 0.2 M HCl and ethanol and then frozen at −24 °C for 24 h. Finally, the aerogels were obtained by freeze-drying the samples using an L4–110 freeze-dryer (GREGOR Instruments, Czech Republic) (Figure S1).

### 2.4 Instrumentation

The molecular weight of BC was calculated by using the Mark–Houwink-Sakurada empirical equation.^27^ BC was first dissolved in 9% lithium chloride/*N,N-*dimethylacetamide solvent system. Relative viscosity was then measured with a Ubbelohde capillary viscometer, which was placed in a water bath tempered at 30 °C.

The BC and BC/PPy composite morphologies were investigated with an MAIA3 Tescan high-resolution scanning electron microscope.

SEM-EDX was performed for the elemental composition and mapping of BC, BC/PPy, and after the adsorption of Cr­(VI) ions and RB using a JEOL JSM-7600F scanning electron microscope equipped with an EDX analyzer Ultim Max from Oxford Instruments, Ltd.

Thermogravimetric analysis of the BC and BC/PPy aerogel was conducted under an air or nitrogen atmospheres at a heating rate of 10 °C min^–1^ up to a temperature of 850 °C using a PerkinElmer Pyris 1 Thermogravimetric Analyzer (USA).

Fourier transform infrared spectra (FTIR) in the region 4000–650 cm^–1^ were recorded using a Thermo Nicolet NEXUS 870 FTIR Spectrometer (MCT/A detector; 256 scans; resolution 2 cm^–1^) equipped with a Golden Gate ATR accessory. The spectra were corrected for the carbon dioxide and humidity in the optical path.

Raman spectra excited with a HeNe 633 nm laser line were recorded with a Renishaw InVia Reflex Raman microspectrometer with a holographic grating of 1800 lines mm^–1^. Spectra excited with a sapphire 488 nm laser were recorded with a Renishaw Qontor Raman microspectrometer with a holographic grating with 2400 lines mm^–1^. A research-grade Leica DM LM microscope was used to focus the laser beam. A Peltier-cooled CCD detector (576 × 384 pixels) registered the dispersed light. Spectra were obtained from 10 spots on each sample, and average spectra are presented (variance spectra are provided in the Supporting Information).

The BET-specific surface area and pore volume of freeze-dried BC and BC/PPy composite were estimated through N_2_ adsorption–desorption isotherms obtained at 77.3 K using a Quantachrome NOVA 2200e analyzer (Quantachrome Instruments, Florida, USA). The samples were degassed at 50 °C for 24 h before measurement.

The UV–visible absorbance spectra of Cr­(VI) and RB solutions were recorded using a Thermo Scientific Evolution 220 UV–visible spectrophotometer (USA).

Inductively coupled plasma mass spectrometry (ICP-MS; NexION 2000 B, PerkinElmer, USA) was employed to quantify the total concentration of Cr­(VI) and Cr­(III) ions in the solutions.

### 2.5 Removal of Hexavalent Chromium Ions

A stock solution of Cr­(VI) ions (500 mg L^–1^) was prepared by dissolving 353.61 mg of K_2_Cr_2_O_7_ powder in 250 mL of Milli-Q water, followed by direct dilutions to prepare the required concentrations. Batch adsorption experiments were conducted by mixing 0.01 g of the BC or BC/PPy composite with 25 mL of Cr­(VI) solutions (25 mg L^–1^). The pH of the solutions was adjusted by adding 0.1 M HCl or NaOH solutions. The adsorption experiments were performed at room temperature (23 ± 1 °C) and a 180 rpm agitation rate under dark conditions. The Cr­(VI) concentration was calculated from their UV–visible absorbance at λ_max_ of 350 nm and an extinction coefficient (ε) of 0.0321 L mg^–1^ cm^–1^ (based on the calibration curve, using Beer–Lambert law). In addition, ICP-MS was employed to quantify Cr­(III) concentration in the solutions at equilibrium.

### 2.6 Removal of Reactive Black 5

A stock solution of RB (200 mg L^–1^) was prepared and directly diluted to the desired concentration in Milli-Q water. Batch adsorption experiments were conducted by mixing 0.01 g of the BC or BC/PPy composite with 25 mL of RB solutions (20 mg L^–1^). The pH of the solutions was adjusted by adding 0.1 M HCl or NaOH solutions. The adsorption experiments were performed at room temperature and a mild shaking rate (180 rpm) in dark conditions. The concentration of RB was quantified from its UV–visible absorbance at λ_max_ of 600 nm and an ε of 0.0351 L mg^–1^ cm^–1^ (based on its calibration curve).

### 2.7 Adsorption Kinetics

The concentrations of Cr­(VI) and RB solutions were monitored over time by measuring their UV–visible spectra. Adsorption capacity (*Q*_t_, mg g^–1^) is expressed as the amount of the adsorbed contaminant per unit mass of the adsorbent and removal efficiency (RE, %) was calculated as the percentage of the contaminant removed from the initial concentration as follows^28^

1 $$Q_{t}=\frac{\left(C_{i}-C_{t}\right)}{m}\times V$$

2 $$\mathrm{RE}=\frac{\left(C_{i}-C_{e}\right)}{C_{i}}\times 100$$

where *C_i_* (mg L^–1^) is the initial concentration of Cr­(VI) or RB, *C_t_* (mg L^–1^) is the concentration of Cr­(VI) or RB at time *t* (min), and *C*_e_ (mg L^–1^) is the concentration of contaminant in the solution at equilibrium, *V* (L) is the volume of the solution and *m* (g) is the mass of the BC or BC/PPy adsorbents.

The adsorption kinetics of Cr­(VI) ions and RB dye were studied within 24 h using the linearized models of pseudo-first-order (eq 3),^29^ pseudo-second-order (eq 4),^30^ and intraparticle diffusion (eq 5)^31^ as follows

3 $$\ln\left(Q_{e}-Q_{t}\right)={\ln Q}_{e}-k_{1}t$$

4 $$t/Q_{t}=1/k_{2}Q_{e}^{2}+t/Q_{e}$$

5 $$Q_{t}=k_{i}t^{1/2}+c_{i}$$

where *Q*_e_ and *Q_t_* (mg g^–1^) are the capacities of Cr­(VI) ions at equilibrium and time *t* (min), respectively, *k*_1_ (min^–1^) is the pseudo-first-order rate constant, and *k*_2_ (g mg^–1^ min^–1^) is the pseudo-second-order rate constant. *k*_*i*_ (mg g^–1^ min^–1/2^) is the intraparticle diffusion rate constant, and *c* (mg g^–1^) is the intraparticle diffusion constant.

### 2.8 Adsorption Isotherms

The equilibrium isotherms were determined for the Cr­(VI) ions and RB dye removal in a batch mode by mixing a constant weight of the BC/PPy (5 mg) with varied initial concentrations of Cr­(VI) or RB solutions (25 mL) in the range of 40–150 or 15–60 mg L^–1^, respectively. The experiments were conducted in the dark at pH 2 for Cr­(VI) or pH 4 for RB solutions, 180 rpm stirring rate, and at room temperature. The equilibrium point was taken after 72 h. To identify the most suitable isotherm model, the experimental data were subjected to fitting using three of the most common equilibrium isotherm models: Langmuir (eq 6),^32^ Freundlich (eq 7),^33^ and Temkin (eq 8),^34^ as follows

6 $$C_{e}/Q_{e}=1/Q_{\max}K_{L}+C_{e}/Q_{\max}$$

7 $${\ln Q}_{e}={\ln K}_{F}+\frac{1}{n}{\ln C}_{e}$$

8 $$Q_{e}=B{\ln K}_{T}+B\ln C_{e}$$

where *Q*_max_ represents the Langmuir maximum amount of adsorbate that can be adsorbed as a monolayer onto the adsorbent surface (mg g^–1^) and *K*_L_ is the Langmuir constant that describes the affinity between the adsorbate and the adsorbent (L mg^–1^). The larger the Langmuir constant value, the stronger the adsorption process. *K*_F_ is the Freundlich constant (mg g^–1^) that represents the adsorption capacity, and *n* is a Freundlich exponent constant that describes the heterogeneity of the adsorption process. The bigger the 1/*n*, the higher the favorability of the adsorption process. *K*_T_ is the Temkin isotherm constant (L g^–1^) that corresponds to the maximum binding energy and *B* is a Temkin constant that describes the heat energy of the adsorption (J mol^–1^).

The correlation coefficient (*R* ^2^) was used to determine the best fitting and validity of the kinetic and isotherm models.

### 2.9 Adsorption/Desorption Cycling

To determine the reusability of the BC/PPy adsorbent, 5 consecutive cycles of adsorption/desorption were performed. The adsorption experiments were performed by mixing 10 mg of BC/PPy composite with 25 mL of 25 mg L^–1^ of Cr­(VI) (pH 2) or 20 mg L^–1^ of RB (pH 4) solutions under dark conditions at 180 rpm. In each cycle, after the adsorption process, the BC/PPy adsorbent was separated and regenerated by soaking in 50 mL of 0.01 M NaOH (pH 12) for 24 h. The adsorbent was then rinsed in a 0.1 M HCl solution before the second adsorption cycle. The removal efficiency of each adsorption cycle was calculated by eq 2.

## 3 Results and Discussion

### 3.1 Characterization

#### 3.1.1 Morphology

Pyrrole was oxidatively polymerized with iron­(III) chloride in the presence of BC (*M*_w_. 433 kDa) hydrogels (Scheme 1). PPy formed a continuous coating layer over the BC fibers through hydrogen bonding and hydrophobic interactions.^35–37^ Figure 1 shows that BC aerogels have a highly porous 3D network structure of unordered, interlaced and smooth cellulose nanofibers with a high aspect ratio and a mean diameter of 36 ± 6 nm (calculated with ImageJ software). BC, after coating with PPy, has maintained its fibrillar structure. However, the fibers get rougher and thicker, with a mean diameter of 149 ± 17 nm. In addition, one can notice that just a few PPy beads were deposited onto BC fiber nodes.

**Figure 1:**
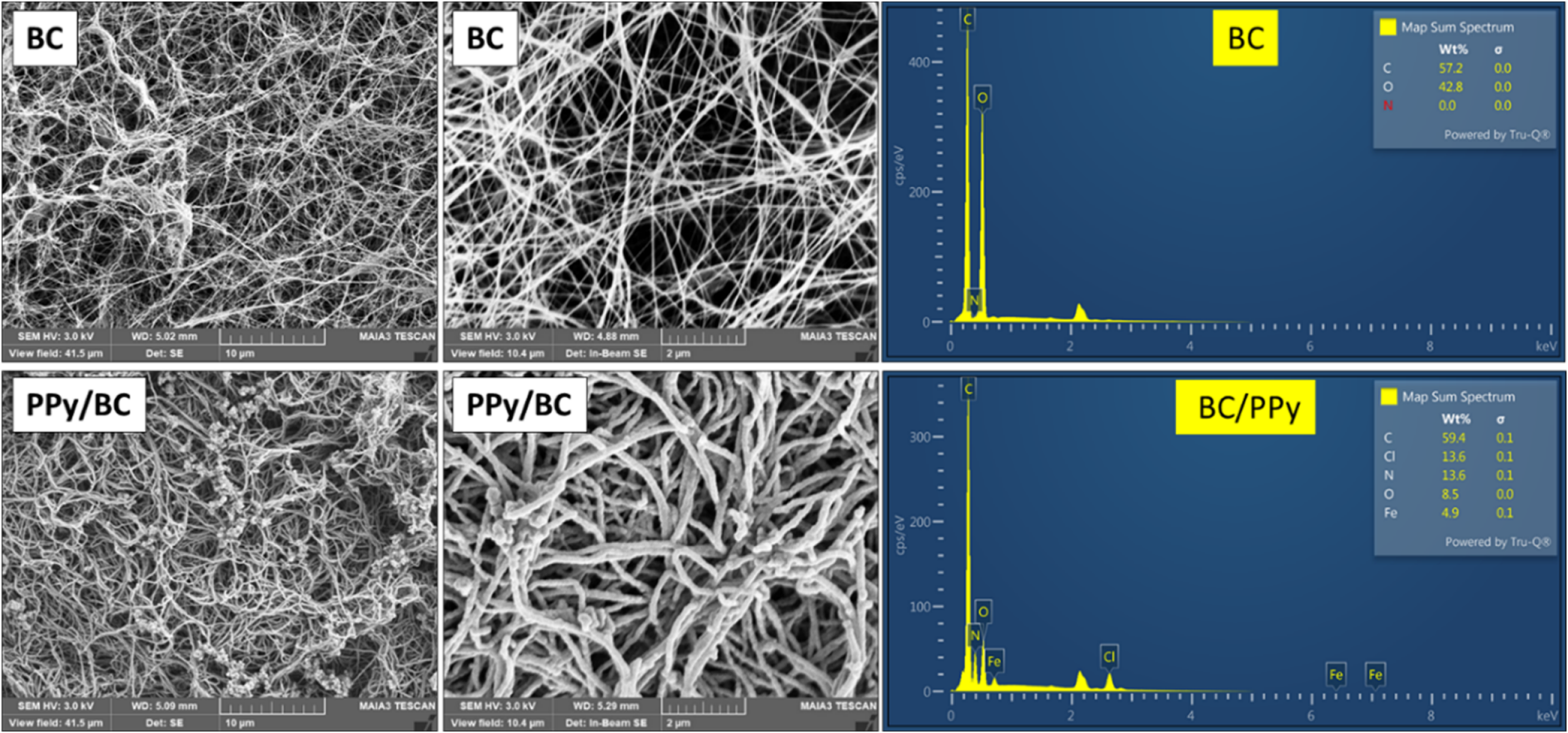
SEM micrographs and SEM-EDX spectra of bacterial cellulose and bacterial cellulose/polypyrrole aerogel.

**Scheme 1:**
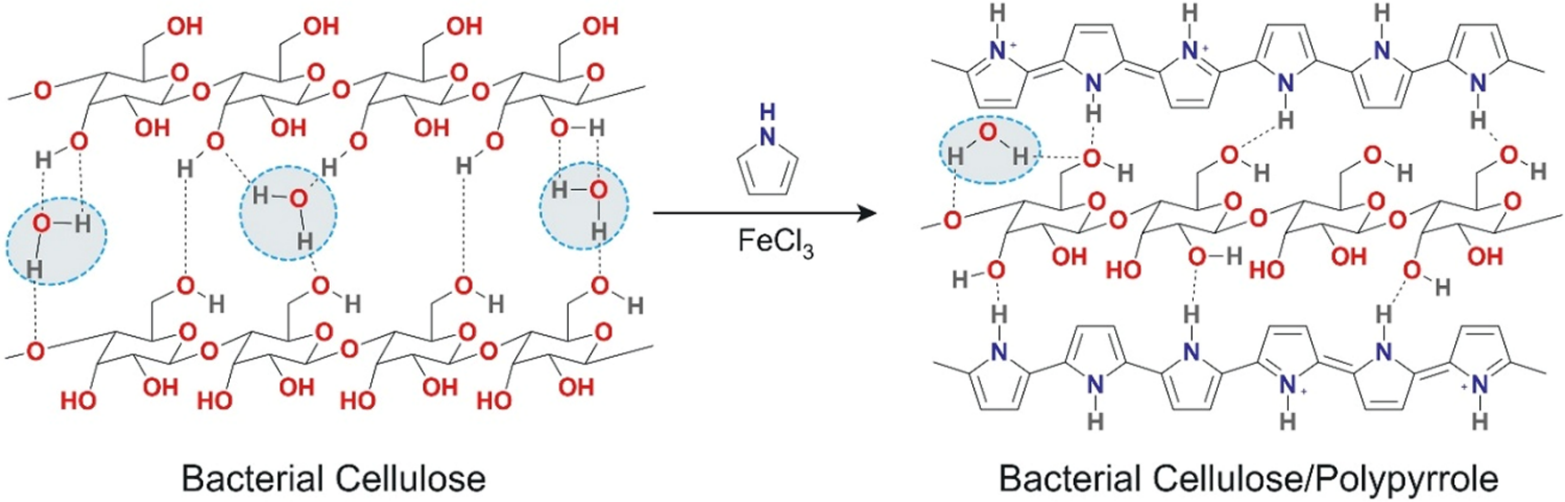
In Situ Oxidative Polymerization of Pyrrole onto Bacterial Cellulose Fibers

The SEM observation is further supported by SEM-EDX analysis. EDX spectra (Figure 1) confirm the successful coating of BC with PPy. In the case of BC, only carbon and oxygen can be detected (hydrogen is a very light element that cannot be detected with EDX). In the case of BC/PPy, the presence of nitrogen (13.6%), a characteristic element of PPy, and chlorine (13.6%) confirms the formation of doped PPy on the BC surface. The presence of Fe in the BC/PPy composite originated from the iron­(III) chloride used as the oxidant for the preparation of PPy. The elemental mapping (Figure S2) reveals the even distribution of C, O, and N elements on the surface of the BC/PPy composite.

#### 3.1.2 Specific Surface Area

The BET surface area of BC and BC/PPy aerogels was calculated to be 56.8 and 38.4 m^2^ g^–1^, respectively. Coating of BC nanofibers with PPy may block the surface pores of the BC fibers, and causing the total pore volume to decrease from 0.091 to 0.067 cm^3^g^–1^. PPy forms a dense layer around the BC fibers, which blocks the pores, increases the overall diameter of the fibers, and reduces the overall accessible surface area and surface-to-volume ratio. As a result, the BET surface area of BC/PPy is smaller than that of neat BC but still is much higher than that of neat globular PPy (13.5 ± 0.4 m^2^ g^–1^).^38^

#### 3.1.3 Thermal Gravimetric Analysis

The thermal stability of BC before and after coating with PPy was investigated in air and under nitrogen. The TGA plots reveal distinct differences between oxidative and inert atmospheres (Figure S3). BC aerogels have less adsorbed water than BC/PPy due to the hygroscopic nature of PPy. In addition, neat BC has more open and less dense networks that reduce the trapped water. Thermal stability improved when BC was coated with PPy. BC fully degraded at 565 °C, while BC/PPy fully degraded at 690 °C. BC was found to fully decompose when treated in air or nitrogen, due to oxidation and pyrolysis processes, respectively.^39^ Unlike the BC, BC/PPy aerogel gets carbonized with 46% char when heated under nitrogen due to its conversion to thermally stable nitrogen-enriched carbonaceous material.^40^

#### 3.1.4 Raman and FTIR Spectroscopy

Vibrational spectroscopy is a useful tool for analyzing the molecular structure of conducting polymers. Raman spectra at 488 nm excitation line and FTIR analysis were used to evaluate the state of PPy and the quality of the formed coating on BC (Figure 2).

**Figure 2:**
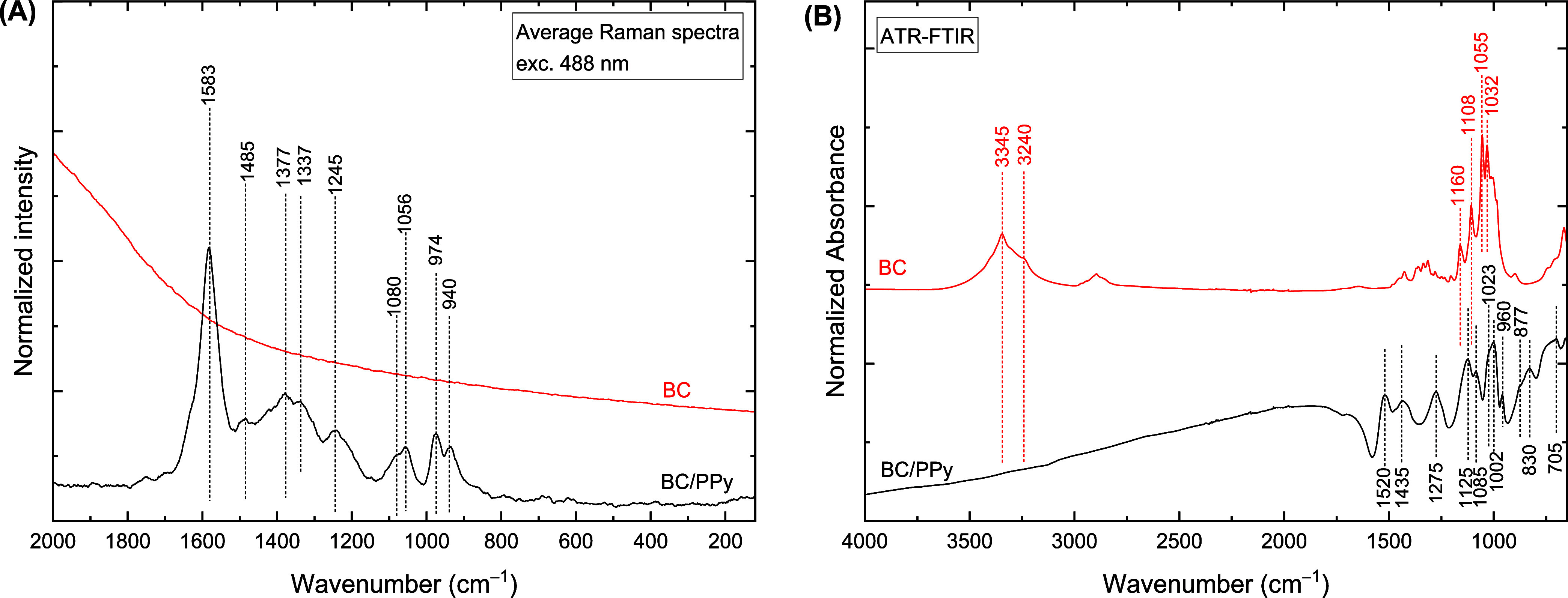
Raman (A) and FTIR (B) spectra of bacterial cellulose and bacterial cellulose/polypyrrole composite.

The Raman spectrum of the BC/PPy composite displays the typical bands of protonated PPy at 1583 cm^–1^ (C=C stretching in charged structures), 1485 cm^–1^ (skeletal vibration), 1377 cm^–1^ (ring-stretching in bipolaron structure) with a shoulder at 1425 cm^–1^ (C—C and C—N stretching), 1337 cm^–1^ (ring-stretching in polaron and neutral structures), 1245 with a shoulder at 1205 cm^–1^ (CH deformation), 1080 cm^–1^ (CH deformation in bipolaron structure), 1056 cm^–1^ (CH deformation in polaron structure), 974 cm^–1^ (ring deformation in polaron structure), and 940 cm^–1^ (ring deformation in bipolaron structure).^41–43^

The FTIR spectrum of the BC/PPy composite consists of bands at 1520 cm^–1^ (pyrrole ring-stretching), 1435 cm^–1^ (C—N stretching), 1275 cm^–1^ (in-plane C—H and C—N deformations), 1125 and 1085 cm^–1^ (C—H and N—H deformations), 1002 with a shoulder at 1023 cm^–1^ (NH^+^ deformation), 960 cm^–1^ (out of plane ring deformation), 877 and 830 cm^–1^ (out of plane CH deformation), and 705 cm^–1^ (out of plane ring deformation).^44,45^ The band positions are shifted compared to the literature due to the distortion associated with the ATR method. Conducting polymers have a high refractive index and the assumption that the ATR-FTIR method is based on is thus not fulfilled. Neither Raman nor FTIR spectroscopy detected spectral responses of BC. This, together with a minimal variance of the Raman spectra (Figure S4), indicates the continuous and homogeneous coating of BC fibers with PPy.

### 3.2 Effect of pH on the Adsorption of Cr­(VI) and RB onto BC/PPy Composite

The adsorption of Cr­(VI) ions is significantly influenced by the pH of the solution, which alters the surface charge of both the adsorbent and the adsorbate.^8^ Depending on the pH of the solution, Cr­(VI) ions can exist in different ionic forms (Figure S5A). H_2_CrO_4_ is formed at pH less than 2.0, HCrO_4_^–^ is the dominant species at pH 2.0–6.0, and CrO_4_^2–^ ions are formed at pH above 6.0.^28,46^ On the other hand, according to the literature, PPy consistently exhibits the highest affinity for Cr­(VI) under acidic conditions (pH 2) due to its protonation and formation of positive charges on its surface that enhance the uptake of Cr­(VI) through electrostatic attraction or ion exchange. Moreover, Cr­(VI) adsorption at low pH was proven to facilitate the electron transfer from electron-rich conducting polymers to Cr­(VI) ions as follows^47^

$$\mathrm{HCr}{O_{4}}^{-}+7H^{+}+3e^{-}⇌{\mathrm{Cr}}^{3+}+4H_{2}O$$

Figure 3A shows that at pH 2, approximately 98% of Cr­(VI) was removed from the solution, and 7.5% of the initial Cr­(VI) concentration was detected as Cr­(III) in the solution by ICP-MS. As the pH of the solution increased, the uptake of Cr­(VI) ions gradually decreased up to pH 4, followed by a significant decrease observed at pH 6, where the removal efficiency dropped to 46.2%. This decrease is attributed to weakened electrostatic interactions and reduced electron transfer capability. At pH 8, the adsorption of Cr­(VI) ions became highly unfavorable, with a removal efficiency of only around 7.5% (Figures 3A and S6). Under basic conditions, Cr­(VI) mainly exists as negatively charged divalent CrO_4_^2–^ species (Figure S5A), and PPy loses its positive charge due to deprotonation.^48^

**Figure 3:**
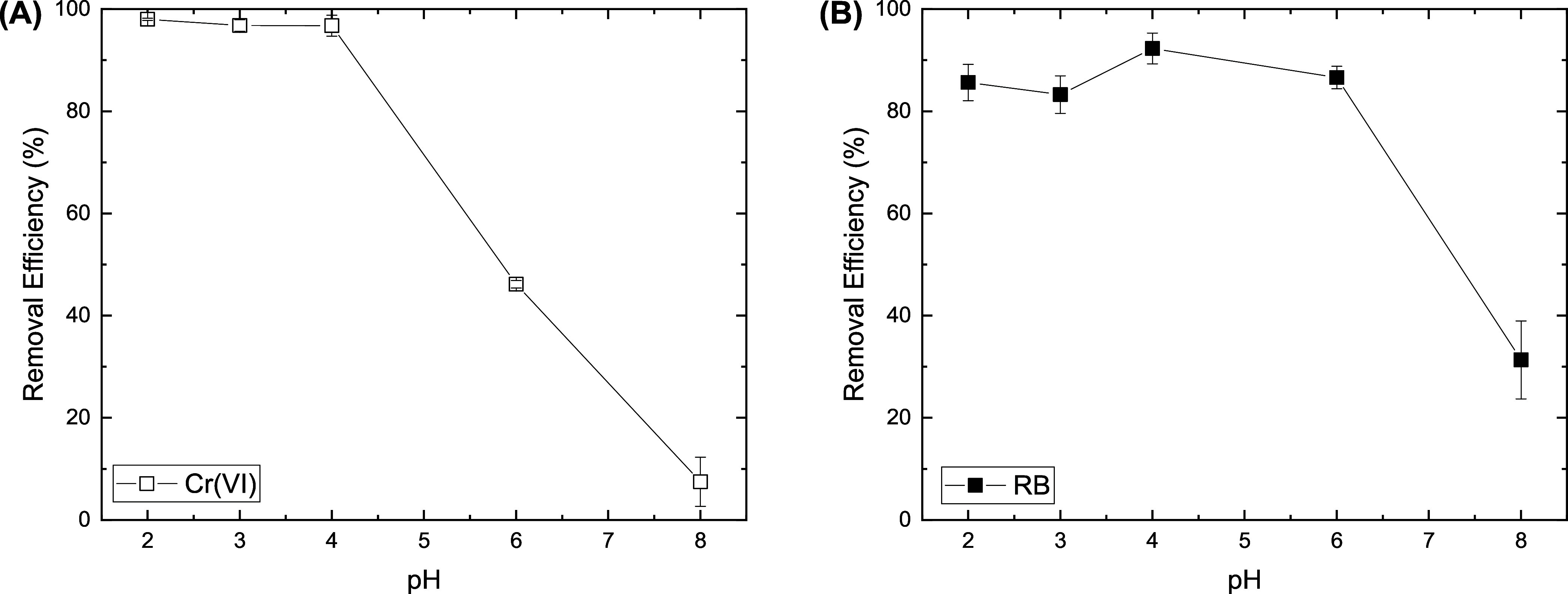
Effect of the initial pH on the adsorption removal efficiency of Cr­(VI) ions (A) and Reactive Black 5 (B) through adsorption onto bacterial cellulose/polypyrrole. Experimental conditions: 5 mg of BC/PPy composite with 25 mL of 60 mg L^–1^ of Cr­(VI), or 20 mg L^–1^ of RB, at a shaking rate of 180 rpm and room temperature.

Similarly, RB (Figure S5B) adsorption onto BC/PPy is significantly influenced by the pH of the solution by altering their surface charges and, consequently, their interactions. Figures 3 and S7 indicate that the highest adsorption efficiency was achieved at pH 4 due to the favorable electrostatic interactions between the ionized sulfonated groups of RB and the positively charged BC/PPy adsorbent.^49^ At pH levels below 4, competition between H^+^ ions and anionic RB may decrease the removal efficiency of the RB. Conversely, at pH 8, a removal efficiency of only 20.2% was achieved due to the deprotonation of the BC/PPy composite.

The maximum removal efficiencies were obtained at pH 2 and 4 for the adsorption of Cr­(VI) and RB, respectively. Therefore, the subsequent adsorption experiments were carried out under these conditions.

### 3.3 Adsorption Kinetics of Cr­(VI) Ions and RB onto BC/PPy

Contact time is one of the main parameters that determines the adsorption efficiency. The effect of contact time on the adsorption efficiency and capacity was evaluated. As expected, BC has a very low affinity for Cr­(VI) ions,^25,35^ showing only a removal efficiency of 4.7% after 24 h (Figure 4). On the other side, BC coated with PPy has demonstrated a high affinity for Cr­(VI) ions with a removal efficiency of 98.6 and 99.6% achieved after 6 and 24 h, respectively. According to the ICP-MS analysis, approximately 10.8% of the initial Cr­(VI) concentration was released to the solution as Cr­(III), which confirms the reduction process. The adsorption equilibrium of RB was achieved after 24 h with a removal efficiency of 23.6 and 99.7% for the neat BC and BC/PPy aerogels, respectively. The results demonstrate a great enhancement of the adsorption rate and efficiency of Cr­(VI) and RB by coating BC with PPy (Figure 5). The fast uptake of the contaminants at the early stage of the adsorption is due to the abundance of easily accessible adsorption sites on the surface of BC/PPy. Over time, these binding sites become occupied, and the adsorbates diffuse into the internal parts of the fibers until the equilibrium is achieved. One can notice that the adsorption kinetics of Cr­(VI) anionic species onto BC/PPy are significantly faster than those of RB. This might be attributed to their smaller molecular sizes and higher effective charge density. The higher effective charges of Cr­(VI) strengthen its electrostatic attraction to the positively charged BC/PPy adsorbent, facilitating faster diffusion and interaction. In contrast, RB has a bulkier structure with a lower effective charge that reduces its diffusion and weakens its interaction.

**Figure 4:**
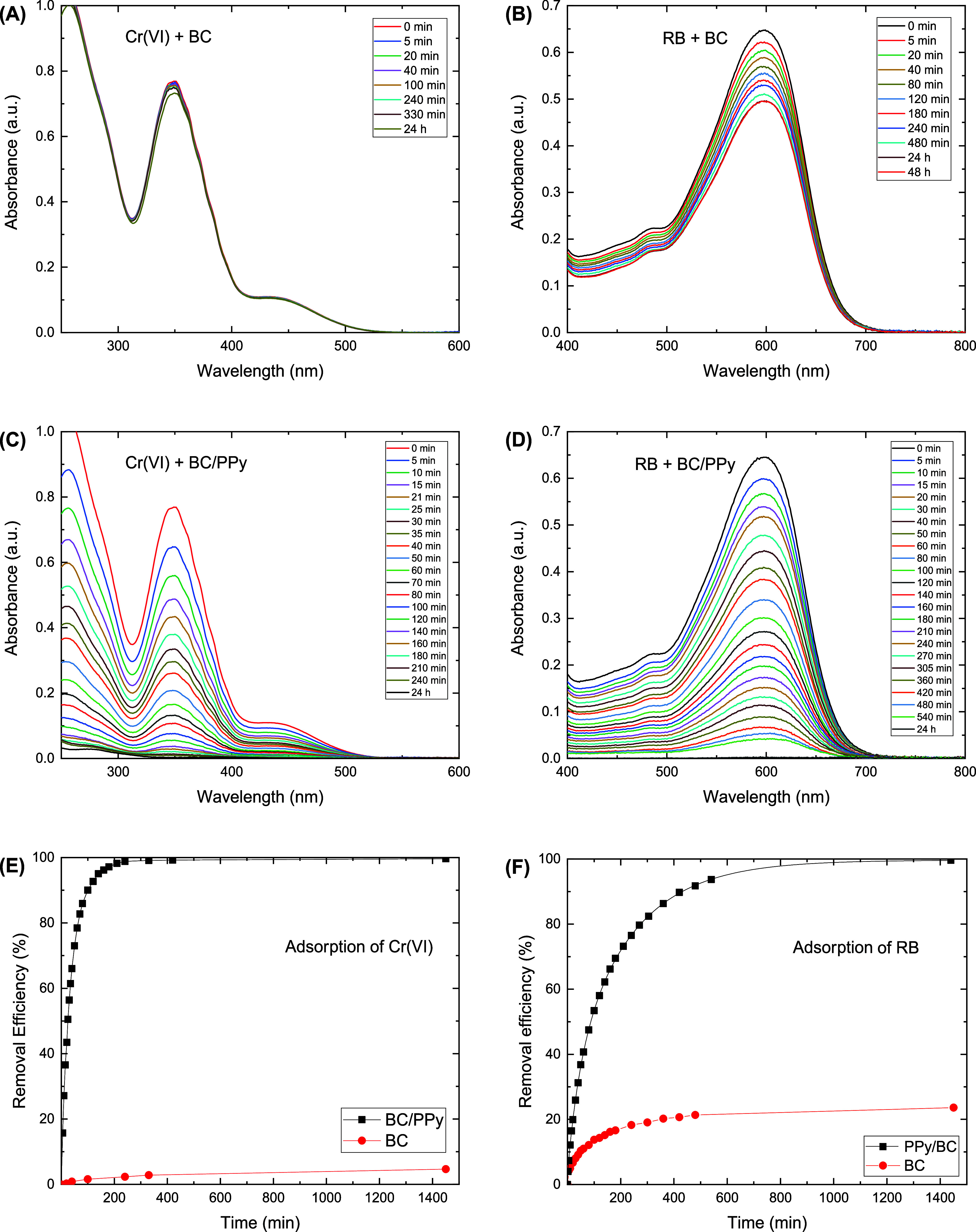
Time-dependence of UV–Vis spectra demonstrating the adsorption of Cr­(VI) ions onto (A) bacterial cellulose, (B) bacterial cellulose/polypyrrole, and the adsorption of Reactive Black 5 onto bacterial cellulose (C) and bacterial cellulose/polypyrrole (D), the effect of adsorption time on the removal efficiencies of Cr­(VI) (E) and Reactive Black 5 (F) over contact time. Experimental conditions: 10 mg of BC/PPy composite with 25 mL of 25 mg L^–1^ of Cr­(VI) at pH 2, or 20 mg L^–1^ of RB at pH 4, at a shaking rate of 180 rpm and room temperature.

**Figure 5:**
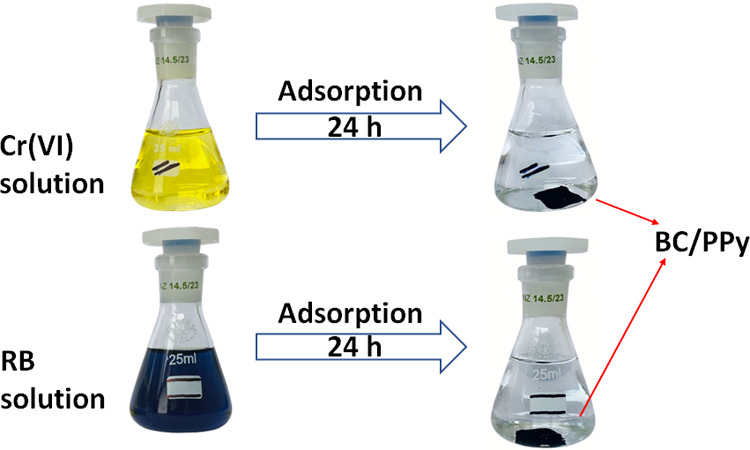
Photographs of Cr­(VI) ions and Reactive Black 5 solutions before and after adsorption onto bacterial cellulose/polypyrrole composite. BC/PPy composite forms stable and easily separated free-standing films that settle down immediately after stopping the shaker.

The time-dependent adsorption profiles of Cr­(VI) and RB onto BC/PPy were further analyzed with the pseudo-first-order, pseudo-second-order, and intraparticle diffusion models (Figure 6). It can be found that the adsorption kinetics of Cr­(VI) and RB were consistent with the pseudo-second-order model, with an *R* ^2^ of 0.999, which is larger than that of the pseudo-first-order model. Furthermore, the theoretically estimated *Q*_e_ values of the pseudo-second-order model are closer to the experimental values (Table 1). This implies that the adsorption process is controlled by chemisorption processes, which could be attributed to the involvement of functional groups (electron-rich hydroxyl and amino groups) of PPy and BC in electrostatic interaction, ion exchange, and redox reactions.

**Table 1:** Adsorption Kinetic Parameters

		Pseudo-first-order			Pseudo-second-order		
adsorbate	*Q*_e_ (exp), mg g^–1^	*Q*_e,_ mg g^–1^	*k*_1,_ min^–1^	*R* ^2^	*Q*_e_, mg g^–1^	*k*_2,_ g mg^–1^ min^–1^	*R* ^2^
Cr(VI)	63.4 ± 0.4	40.07	0.0161	0.936	62.2	0.00095	0.999
RB	51.2 ± 1.8	43.6	0.0052	0.989	54.8	0.00021	0.999

**Figure 6:**
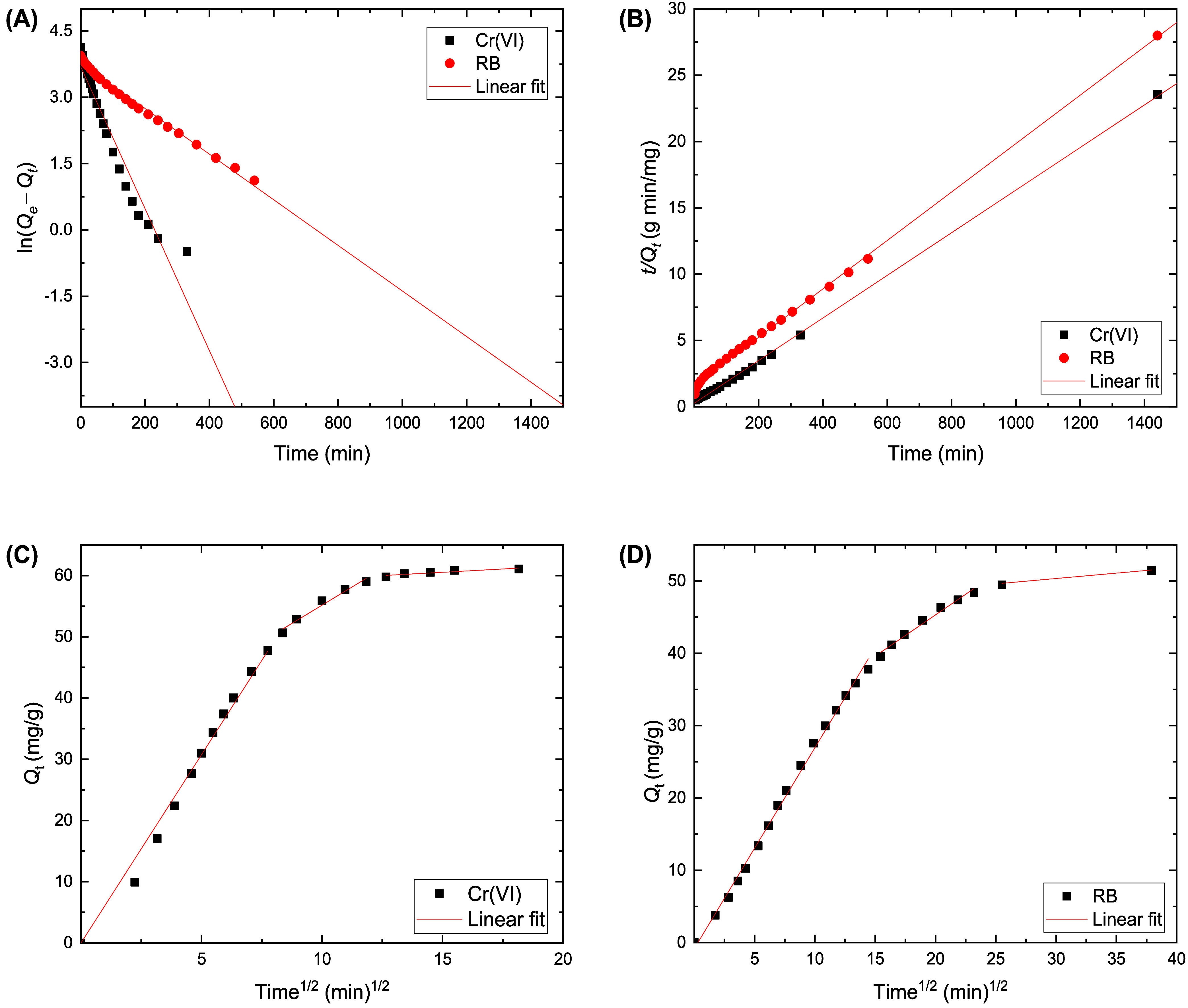
Pseudo-first-order (A) and pseudo-second-order (B) kinetics modeling, and intraparticle diffusion modeling for Cr­(VI) ions (C) and RB (D) adsorption.

The plots of intraparticle diffusion (Weber-Morris) modeling were used for a better understanding of the diffusion processes of the adsorbates (Cr­(VI) ions and RB) onto the BC/PPy adsorbent. As depicted in Figure 6C,D, the plots of *Q*_t_ versus *t* ^1/2^ can be divided into three stages: (1) fast initial adsorption onto the surface (with the highest rate stage), (2) a gradual decrease in adsorbate concentration, where the intraparticle diffusion plays a role (slower rate stage), and (3) the equilibrium stage. The second section indicates that intraparticle diffusion is the rate-limiting step in the adsorption processes of Cr­(VI) and RB, with *k_i_* values of 2.39 and 1.6 mg g^–1^ min^–1/2^ and intercepts of 31.3 and 22.5 mg g^–1^, respectively.

### 3.4 Equilibrium Isotherms of Cr­(VI) Ions and RB

The effect of the initial concentrations of Cr­(VI) ions or RB contaminants was found to control the removal efficiency (%) as well as the adsorption capacity (*Q*_e_), as shown in Figure 7.

**Figure 7:**
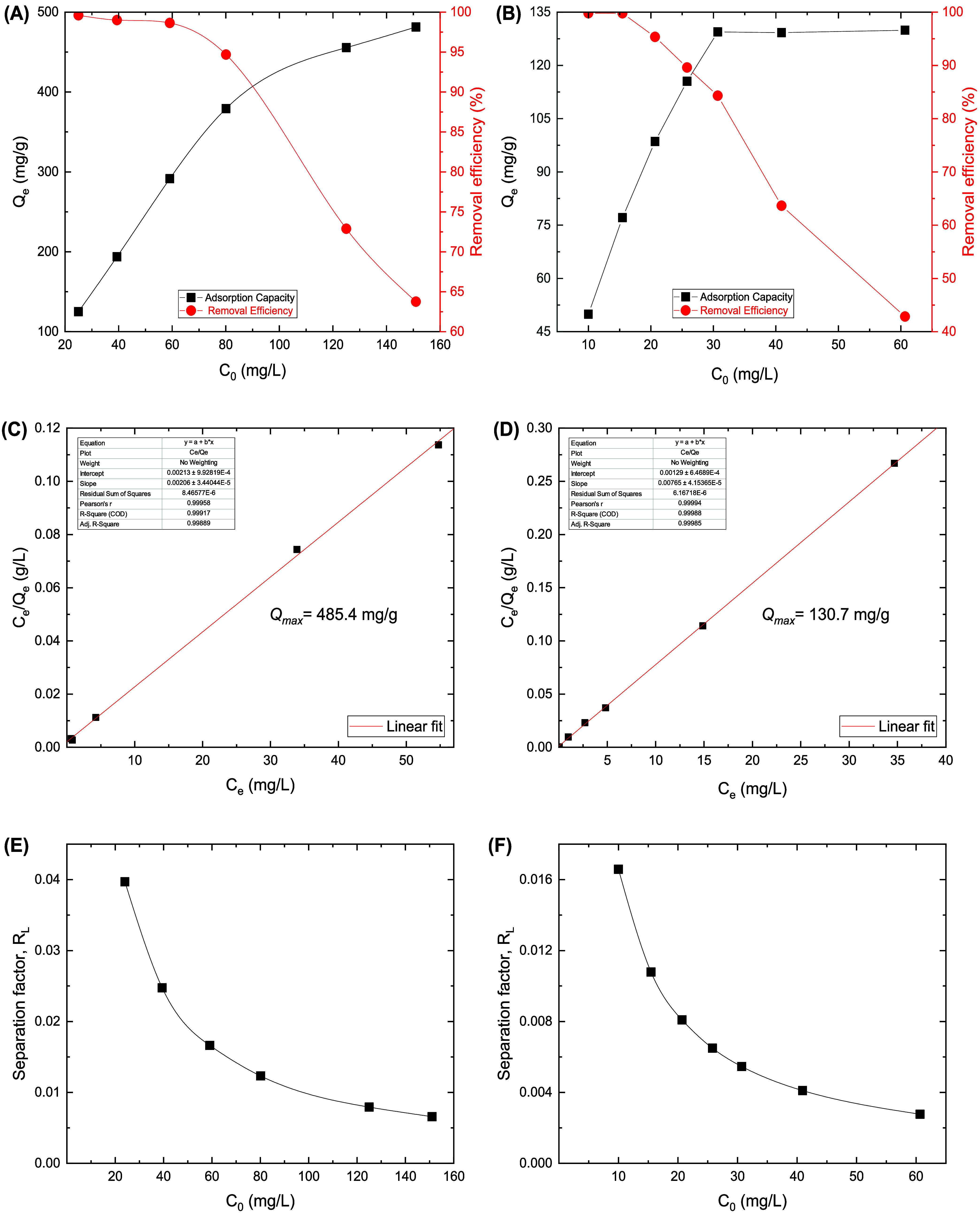
Effect of initial concentrations on the adsorption capacity and removal efficiency of Cr­(VI) (A) and Reactive Black 5 (B). Langmuir isotherm modeling of Cr­(VI) (C) and Reactive Black 5 (D) adsorption. Langmuir separation factor of Cr­(VI) (E) and Reactive Black 5 (F) adsorption.

Increasing the initial concentration of Cr­(VI) ions increases the adsorption capacity, and a maximum adsorption capacity of 481.3 mg g^–1^ was achieved at the initial concentration of 150 mg L^–1^. At the same time, the removal efficiency decreases due to the limited binding sites that become saturated at higher Cr­(VI) concentrations. In a similar manner, Figure 7B shows that removal efficiency % above 99% was achieved for the RB concentrations of 10 and 15 mg L^–1^. Further increases in the initial concentrations of RB (20–60 mg L^–1^) led to a gradual decrease in the removal efficiency. At the same time, the adsorption capacity increased with increasing the initial concentration of RB in the solution up to a maximum value of 130.2 mg g^–1^. These adsorption results from initial concentration-dependent experiments were used to investigate the adsorption mechanism by studying adsorption isotherms. Figure 7C,D reveal that the Langmuir isotherm model fits the empirical data perfectly (*R* ^2^ = 0.999) with a Langmuir maximum adsorption capacity of 485.4 mg g^–1^ for Cr­(VI) ions and 130.7 mg g^–1^ for RB (Table 2), which are very close to the experimental results (Figure 7). This implies that the adsorption of Cr­(VI) ions and RB molecules takes place as a homogeneous monolayer onto BC/PPy fibers, with no interaction between the adsorbed molecules on the neighboring binding sites.^14^ The Langmuir model can additionally be expressed as a dimensionless constant called the separation factor (*R*_L_) as follows^50^

9 $$R_{L}=1/\left(1+K_{L}C_{0}\right)$$

**Table 2:** Equilibrium Isotherm Parameters

		Langmuir			Freundlich			Temkin		
adsorbate	*Q*_max_ (exp), mg g^–1^	*Q*_max_, mg g^–1^	*K*_L,_ L mg^–1^	*R* ^2^	*K*_*F*,_ mg g^–1^	1/*n*	*R* ^*2*^	*K*_*T*,_ L g^–1^	*B*, J mol^–1^	*R* ^*2*^
Cr(VI)	481.3	485.4	0.97	0.999	260.3	0.137	0.842	190.6	52.43	0.941
RB	130.2	130.7	5.93	0.999	103.5	0.083	0.926	2.5 × 10^5^	8.51	0.917

*R*_L_ values indicate the adsorption process to be unfavorable (*R*_L_ > 1), linear (*R*_L_ = 1), favorable (0 < *R*_L_ < 1), or irreversible (*R*_L_ = 0).^51^ The lower the *R*_L_ value, the more favorable the adsorption process. Figure 7E,F reveal that *R*_L_ values range from 0.04 to 0.006 for Cr­(VI) and 0.017 to 0.003 for RB adsorption, indicating a highly favorable adsorption process of Cr­(VI) and RB species onto BC/PPy at the used different initial concentrations of Cr­(VI) and RB solutions.

Additionally, the Freundlich and Temkin isotherm models were used to fit the experimental data (Figure S8). The Freundlich isotherm model assumes that adsorption takes place on a heterogeneous surface by multilayer adsorption. The Temkin isotherm model assumes that the adsorption is characterized by a uniform distribution of binding energies up to some maximum coverage and the heat of adsorption decreases linearly with coverage due to adsorbent–adsorbate interactions. Both Cr­(VI) and RB adsorption processes have shown poor fitting with the Freundlich and Temkin models (Figure S8and Table 2).

### 3.5 Reusability of BC/PPy

The reusability of BC/PPy adsorbent for removing Cr­(VI) ions and RB was examined by consecutively performing adsorption/desorption processes (five cycles), using 20 and 25 mg L^–1^ of RB and Cr­(VI) solutions, respectively. Figures 8A and S9 reveal that BC/PPy can effectively adsorb Cr­(VI) ions over multiple cycles. However, the removal efficiency of Cr­(VI) ions gradually decreased from 99.6% in the first cycle to 92.1% in the fifth cycle. This may be attributed to the partial degradation of the polymeric chains by the oxidizing Cr­(VI) solutions as discussed below.^52^ On the other side, RB can be removed with minimal change in the efficiency across the regeneration cycles. The removal efficiency of 99.7% in the first cycle decreased slightly to 97.7% in the fifth cycle (Figures 8B and S10).

**Figure 8:**
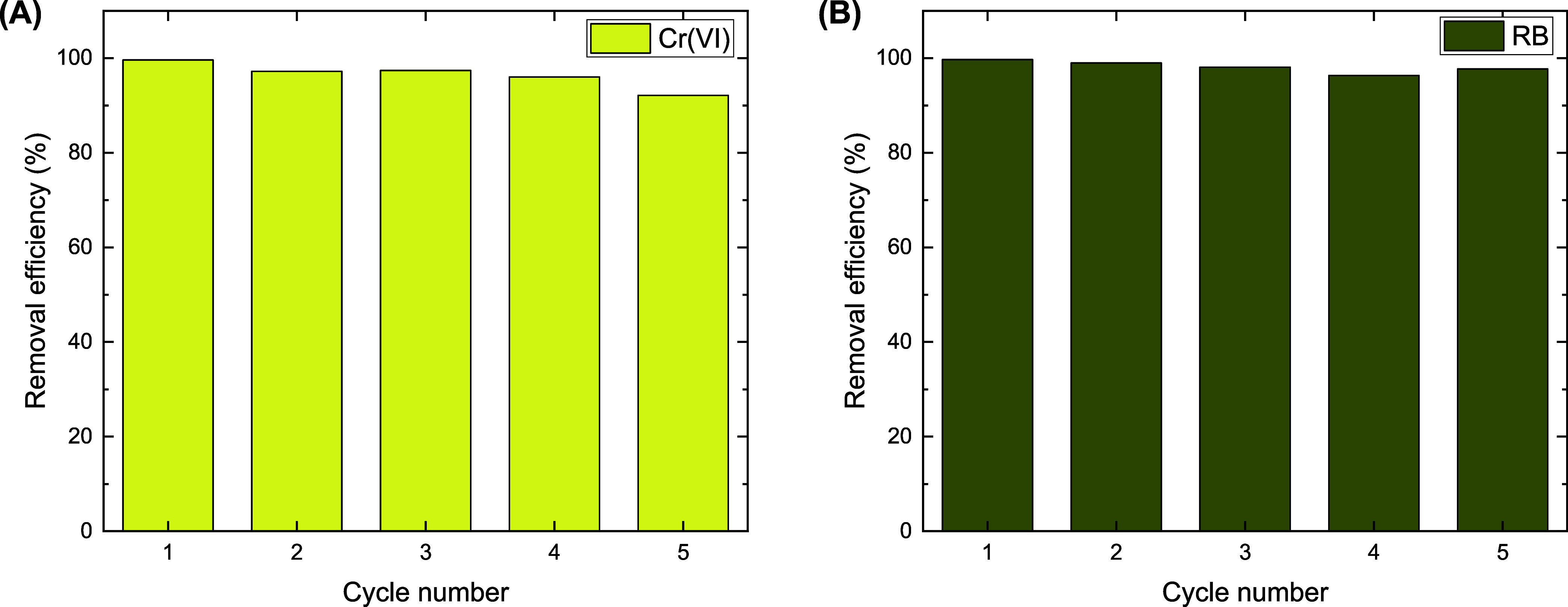
Removal efficiencies of Cr­(VI) ions (A) and Reactive Black 5 (B), evaluated across different adsorption/desorption cycles. Experimental conditions: 10 mg of BC/PPy composite with 25 mL of 25 mg L^–1^ of Cr­(VI) at pH 2, or 20 mg L^–1^ of RB at pH 4, at a shaking rate of 180 rpm and room temperature.

### 3.6 Adsorption Mechanism

#### 3.6.1 Elemental Mapping

SEM-EDX mapping was employed to identify the elements of Cr and S after the adsorption of Cr­(VI) and RB, respectively. The determined atomic content of Cr and S atoms was found to be 10 and 3%, respectively, based on the quantitative analysis of EDX-elemental mapping (Figure 9). This indicates the effective removal of Cr­(VI) and RB by BC/PPy aerogels. Moreover, Figure 9 illustrates the uniform distribution of Cr and S atoms, indicating the even binding of Cr­(VI) ionic species and RB molecules onto the BC/PPy aerogel. Further, the distribution of Cr and S elements follows a similar pattern to that of the N element, which may indicate that the pyrrolic N functional groups are the main binding sites for adsorption. Furthermore, a comparison of the elemental composition of BC/PPy before and after the adsorption of Cr­(VI) or RB (Figures 1 and 9) reveals that (1) after the adsorption of Cr­(VI), the oxygen content has increased nearly 3-fold, while nitrogen content remained almost unchanged, (2) after RB adsorption, the content of oxygen and nitrogen remained unchanged, (3) In both adsorption cases, a decrease in Cl ion content was observed. These findings suggest that the PPy layer coating BC underwent oxidation, accompanied by ion exchange between Cl ions and Cr­(VI) anionic species, whereas for RB, only ion exchange occurred.

**Figure 9:**
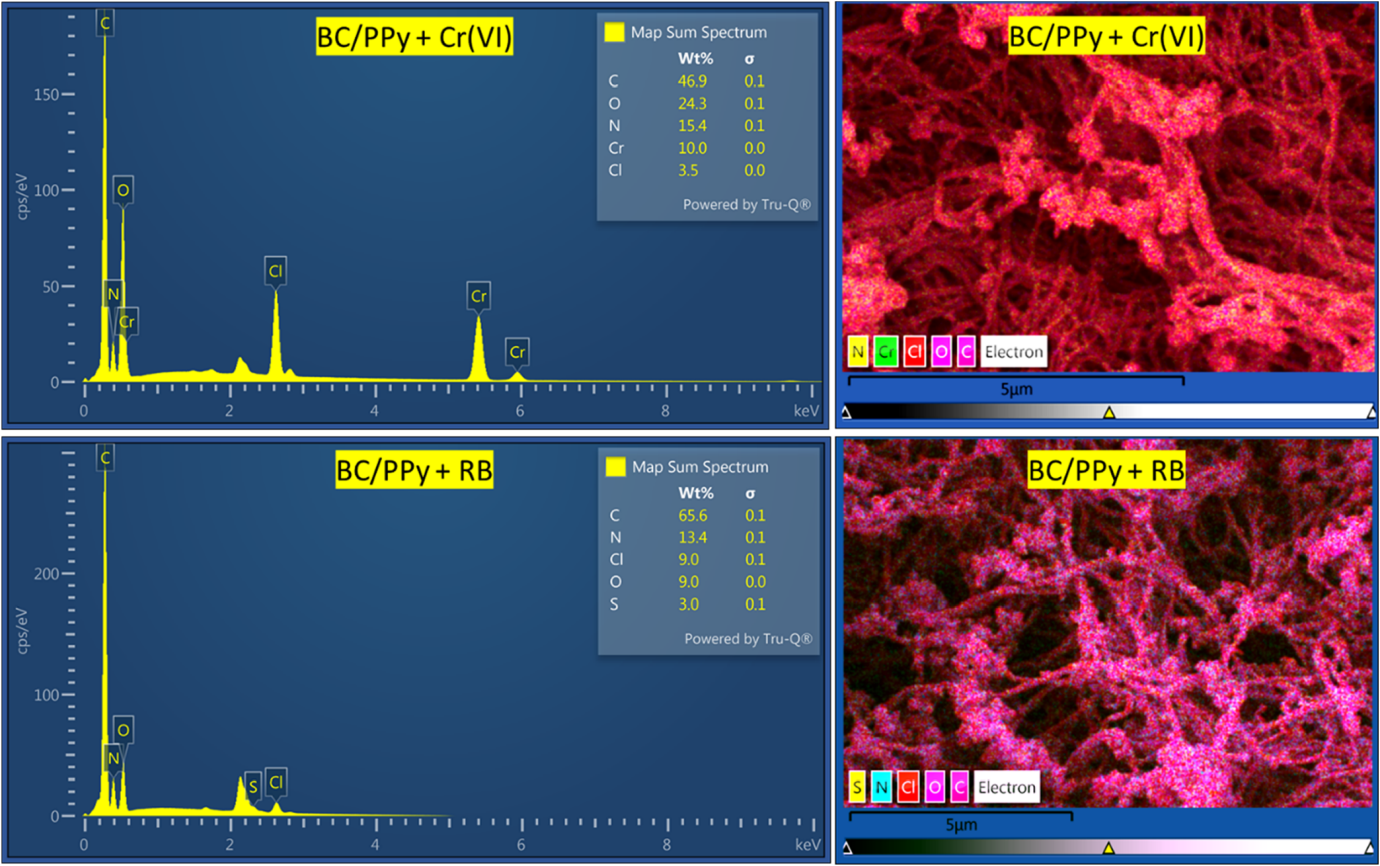
EDX spectra of bacterial cellulose/polypyrrole composite after the adsorption of Cr­(VI) ions and Reactive Black 5 (RB), and their elemental mapping.

#### 3.6.2 Raman and FTIR Spectroscopy

Even though Cr­(VI) ions were not directly detected by vibrational spectroscopy (Figures 10 and S11), their impact on the BC/PPy substrate was. The Raman spectrum of BC/PPy after the adsorption of Cr­(VI) ions (Figure 10A) showed that the band at 1583 cm^–1^ broadened, the intensity of the band at 1425 cm^–1^ increased, while the bands connected with bipolarons at 1377, 1245, 1080, and 940 cm^–1^ almost disappeared. Furthermore, several bands shifted toward positions typical of neutral structures^43^ (1583 → 1595 cm^–1^, 1056 → 1048 cm^–1^, 974 → 988 cm^–1^), and a substantial shoulder appears around 1545 cm^–1^ (C=C stretching in neutral structures).^43^ These changes are related to the deprotonation of PPy. In addition, the charge-insensitive CH deformation bands at 1245 and 1205 cm^–1^ decreased significantly, indicating the degradation of the PPy chains through oxidation, cross-linking, or other functionalization of the pyrrole ring.

**Figure 10:**
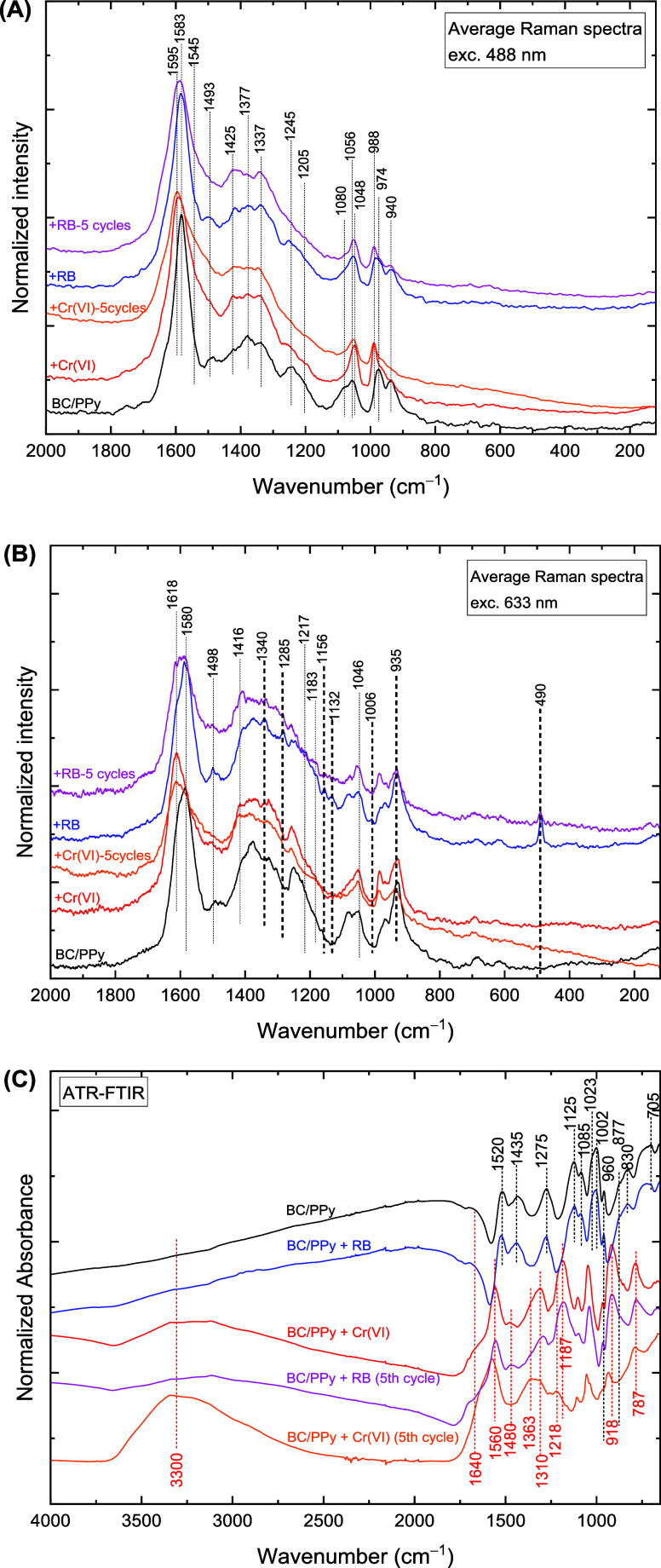
Raman spectra of bacterial cellulose/polypyrrole, after adsorption of Cr­(VI) ions and Reactive Black 5 (RB), and after 5 adsorption/desorption cycles, excited at 488 nm (A) and 633 nm (B). In panel B, Raman bands associated solely with RB are marked with thicker lines. FTIR spectra of bacterial cellulose/polypyrrole composite before and after the adsorption of Cr­(VI) and RB, and after 5 adsorption/desorption cycles (C).

The FTIR spectrum of the BC/PPy substrate exposed to Cr­(VI) solution (Figure 10C) displays features of a PPy base at 1560 cm^–1^ (pyrrole ring-stretching), 1480 cm^–1^ (C—N stretching), 1310 cm^–1^ with a shoulder at 1363 cm^–1^ (in-plane C—H and C—N deformations), 1187 cm^–1^ with a shoulder at 1218 cm^–1^ (C—H and N—H deformation), 1040 cm^–1^ (C–H and N–H in-plane deformation, cellulose), and 918 and 787 cm^–1^ (out of plane CH deformation).^42,44^ The small peak at 1108 cm^–1^ is attributed to the BC substrate. The broad band around 3300 cm^–1^ and a shoulder around 1640 cm^–1^ are due to water adsorbed or serving as a ligand in a complex.^53^ In addition, broad bands can be detected around 1705 and 1630 cm^–1^ (C=O stretching in COOH), 1590 cm^–1^ (COO^–^ antisymmetric stretching), 1360 cm^–1^ (COO^–^ symmetric stretching), 1330 cm^–1^ (O–H deformation in COOH), 1190 cm^–1^ (C–O stretching in COOH), and 920 cm^–1^ (COO^–^ deformation).^54^ These bands indicate oxidative degradation of PPy.^42,55^ The deprotonation of PPy (due to the increased local pH), the presence of a high amount of bound water, and the oxidative degradation of PPy support the adsorption mechanism of Cr­(VI) ions that include the reduction of Cr­(VI) to Cr­(III).^21^ The Cr­(III) ions then form complexes with water, chloride ions, and the newly formed carboxylate ions.^53^

The Raman spectrum of BC/PPy after the adsorption of RB, excited at 488 nm (Figure 10A), shows only slight changes: The band at 1583 cm^–1^ slightly broadens, the intensity of the band at 1425 cm^–1^ increases, and the bands associated with the bipolarons at 1377, 1245, 1080, and 940 cm^–1^ decrease. These changes indicate a shift in the balance of charge carriers from bipolarons to polarons. RB was resonantly enhanced and identified using a 633 nm excitation line (Figure 10B). All RB band positions are consistent with those reported in the literature,^56,57^ which indicates the absence of specific interactions such as π–π interactions or H-bonding. Additionally, the variance spectra (Figure S4) are very weak and practically featureless, which indicates high homogeneity of the samples, including homogeneous adsorption of RB throughout the sample volume.

The FTIR spectra of the BC/PPy before and after the adsorption of RB are practically identical (Figure 10C), indicating that PPy remains protonated. The absence of band shifts indicates against specific interactions. Although PPy remains protonated and nondegraded, the ratio between bipolarons and polarons decreases. These observations suggest that RB partially dopes PPy. As a larger molecule compared to the original doping ions (chlorides), RB is sterically hindered and unable to stabilize bipolarons.

The effect of adsorption/desorption cycling of Cr­(VI) on the stability of the BC/PPy substrate was also studied. Figure 10A shows that the Raman spectra of BC/PPy after single Cr­(VI) adsorption and after adsorption/desorption cycling of Cr­(VI) ions are essentially identical, except for the complete disappearance of the C—H stretching bands at 1245 and 1205 cm^–1^, and the bands are broader and less defined. The FTIR spectra of the cycled BC/PPy display broad bands around 1600, 1363, and 940 cm^–1^, connected with carboxylic groups formed due to the alkaline oxidation of PPy. The oxidative degradation of the BC/PPy adsorbent is thus more prominent due to extended exposure to high pH conditions during repeated Cr­(VI) exposure and alkaline washing.^55^

The Raman spectrum of BC/PPy after adsorption/desorption cycling shows a peak at 490 cm^–1^ assigned to residual RB (Figure 10B), indicating that the RB was not fully desorbed. The FTIR spectrum of BC/PPy after adsorption/desorption cycling of RB (Figure 10C) is very similar to the spectrum of BC/PPy with adsorbed Cr­(VI), differing in the amount of adsorbed water. The repeated alkaline washing thus induced some oxidative degradation in the sample.

### 3.7 Adsorption of a Binary Mixture of Cr­(VI) and RB onto BC/PPy Composite

The adsorption of a binary mixture of Cr­(VI) and RB was conducted at the initial concentrations of 25 and 20 mg L^–1^, respectively. The adsorption of the binary mixture was carried out at pH 4, as this pH level provides optimal conditions for the adsorption of both Cr­(VI) and RB (discussed in Section 3.2). Unlike the single solute systems, the adsorbent/adsorbate affinity may change due to the competition for available binding sites.^58^ Figure 11 shows that about 99.6% of RB and 99.0% of Cr­(VI) ions were removed from the solution within 24 h. The total adsorption capacity (*Q*_T,_ mg g^–1^) in a multicomponent system can be calculated as follows^59^

10 $$Q_{T}=\sum_{i=1}^{n}Q_{i}$$

where *Q*_*i*_ is the adsorption capacity of component *i* and *n* is the number of components. The total adsorption capacity was estimated to be 223.4 mg of Cr­(VI) and RB per gram of BC/PPy composite.

**Figure 11:**
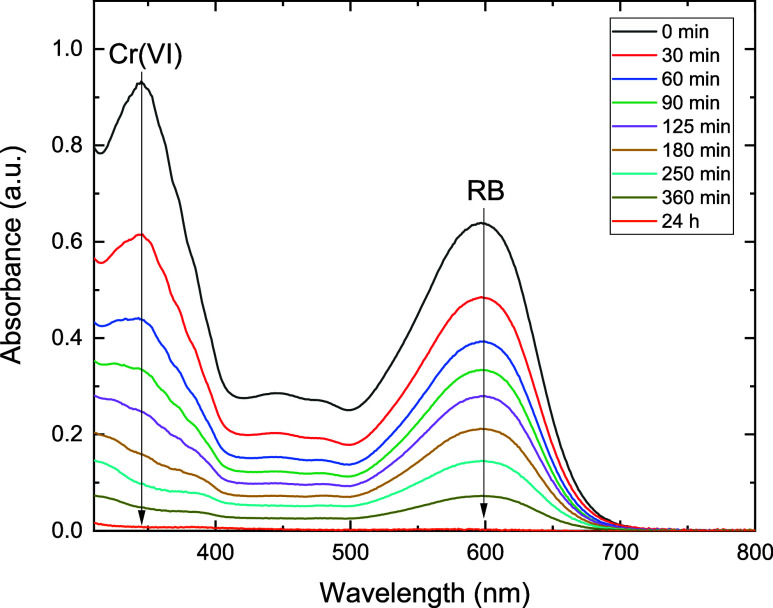
Time-dependence of UV–Vis spectra demonstrating the adsorption of a binary mixture of Reactive Black 5 and hexavalent chromium ions onto bacterial cellulose/polypyrrole adsorbent.

The adsorption of a binary mixture of Cr­(VI) and RB not only simulates the conditions of real wastewater containing multiple pollutants but also appears to be less damaging to the BC/PPy adsorbent compared to the adsorption of Cr­(VI) ions alone. The FTIR and Raman spectra of the substrate with combined adsorption of Cr­(VI) and RB are very similar to the spectra of the neat BC/PPy substrate and BC/PPy with RB only (Figures 10 and S11B). No sign of oxidative degradation was detected. This indicates that RB may protect the substrate from oxidation by Cr­(VI). Furthermore, the elemental composition of BC/PPy shows that the oxygen content increases less when a binary mixture of Cr­(VI) and RB was adsorbed (Figure S12), compared to Cr­(VI) alone (Figure 9), suggesting reduced oxidative effect. The reduced oxidation makes the adsorption of a binary mixture more advantageous.

## 4 Conclusions

Bacterial cellulose hydrogels were coated with PPy through an in situ polymerization of pyrrole with iron­(III) chloride as an oxidant. PPy was uniformly deposited onto BC fibers, resulting in an increase in the BC fibers’ thickness from 36 ± 6 to 149 ± 17 nm. Even though coating of BC with PPy has reduced the surface area, it has introduced functionality and conductivity. Additionally, thermogravimetric analysis revealed an enhancement in the thermal stability of BC after coating with PPy. BC/PPy aerogels form stable, easily separable free-standing films that settle immediately once shaking is stopped, enabling convenient separation of the adsorbent from the solution without leaving any suspended particles. The adsorption experiments showed a significant enhancement of the adsorption capacity of Cr­(VI) ions and RB onto the BC/PPy composite, as compared to neat BC. The pseudo-second-order kinetics and Langmuir isotherm models well described the single solute adsorption of Cr­(VI) ions as well as RB. According to the Langmuir model, the maximum adsorption capacities of 485.7 and 130.7 mg g^–1^ of Cr­(VI) and RB, respectively, were achieved for the BC/PPy composite. FTIR and Raman spectroscopies indicate that the adsorption of Cr­(VI) ions onto BC/PPy is due to ion exchange and involves electron transfer between PPy and Cr­(VI) ions to form Cr­(III) ions. The adsorption of RB is mainly due to ion exchange with Cl^–^ ions (the PPy dopant). The BC/PPy adsorbent has demonstrated high stability, achieving removal efficiencies of 92.1% for Cr­(VI) ions and 97.7% for RB after 5 adsorption/desorption cycles. The decrease in efficiency for the Cr­(VI) ions removal with cycling is attributed to the degradation effect of Cr­(VI) on PPy chains through oxidation. The adsorption of a binary mixture of Cr­(VI) and RB has a less oxidative effect on the BC/PPy adsorbent compared to the adsorption of Cr­(VI) alone.

## Supplementary Material



## Notes

Photographs of bacterial cellulose hydrogel and freeze-dried bacterial cellulose/polypyrrole; C, O, N and Cl elemental mapping; TGA analysis; variance Raman spectra; Speciation of Cr­(VI) ions; molecular structure of Reactive Black 5; effect of pH on the removal efficiency of Cr­(VI) (60 mg/L, 25 mL) and RB (20 mg/L, 25 mL); Freundlich and Temkin isotherm modeling of Cr­(VI) and RB adsorption; Cr­(VI) UV−Vis spectra at different cycles; RB UV−Vis spectra at different cycles; FTIR spectra of BC, BC/PPy with adsorbed RB and Cr­(VI); EDX spectra and elemental mapping of the BC/PPy composite after adsorption of a binary mixture (PDF)
