# Supplementary material for: Bacterial Cellulose/Polypyrrole Aerogel for the Efficient Removal of Organic and Inorganic Water Contaminants
Source: ACS Omega. 2026 Apr 8;11(15):23359–73. doi: 10.1021/acsomega.6c00432 (PMC13103771; doi:10.1021/acsomega.6c00432)
Supplement: Supplementary file 1 [file ao6c00432_si_001.pdf]

Supporting Information

Bacterial cellulose/polypyrrole aerogel for the efficient removal of organic and inorganic water contaminants

Islam M. Minisy[1], Radim Striz[2], Zuzana Moravkova[1], Adriana Kovalcik[2], Patrycja Bober[1,*]

[1]Institute of Macromolecular Chemistry, Czech Academy of Sciences, 162 00 Prague, Czech Republic

[2]Faculty of Chemistry, Brno University of Technology, Purkynova 118, 612 00 Brno, Czech Republic

[*] Corresponding author, e-mail address: bober@imc.cas.cz (P. Bober)

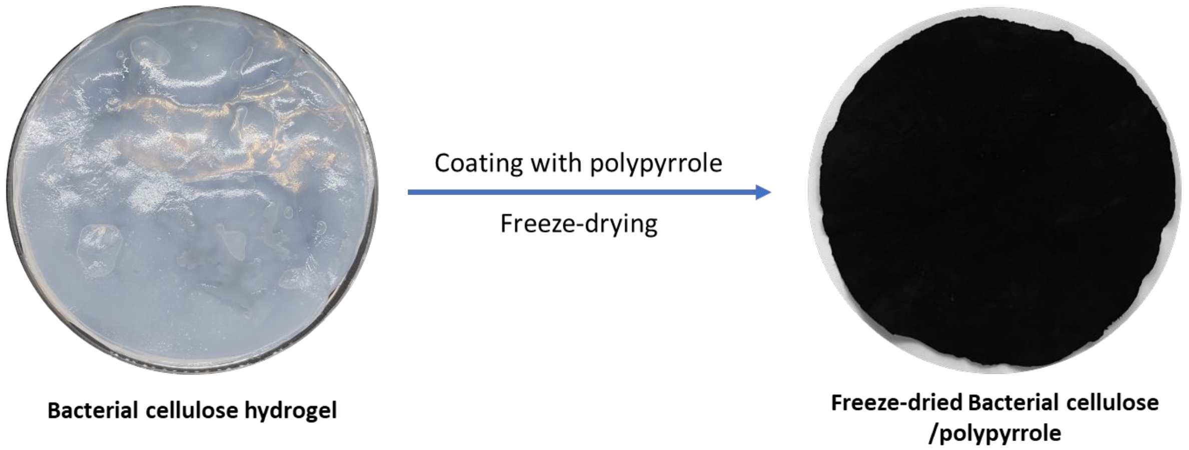

Fig. S1. Photographs of bacterial cellulose hydrogel and freeze-dried bacterial cellulose/polypyrrole.

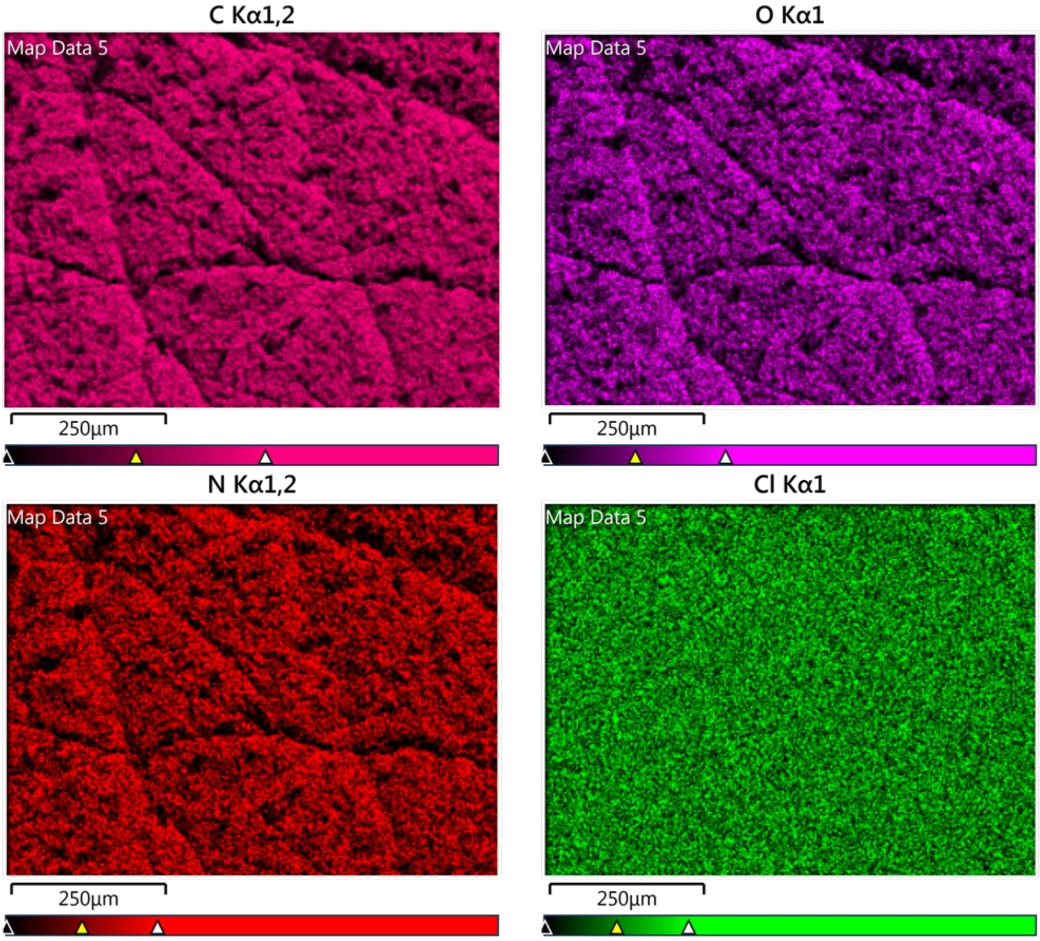

Fig. S2. Carbon, oxygen, nitrogen, and Cl elemental mapping of BC/PPy composite.

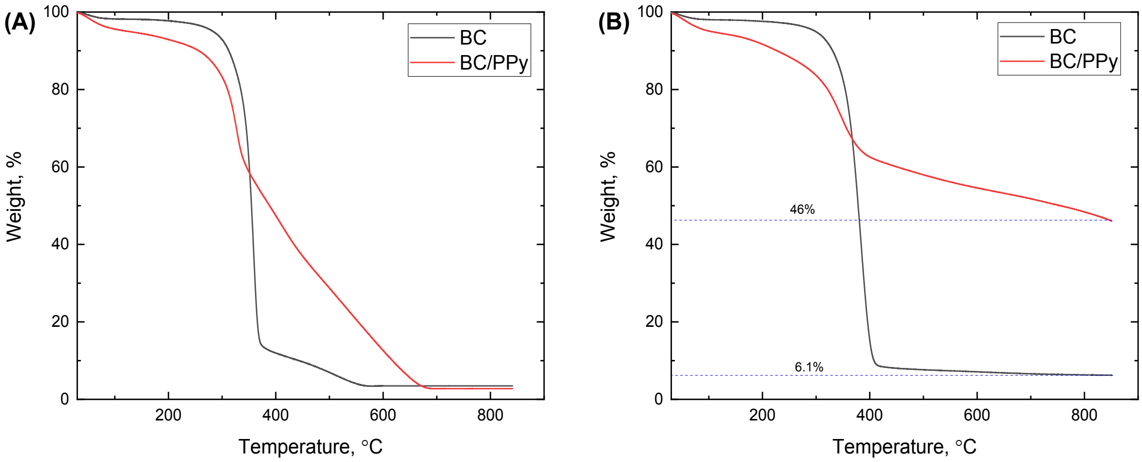

Fig. S3. Thermal gravimetric analysis of bacterial cellulose and bacterial cellulose/polypyrrole in air (A) and under nitrogen (B).

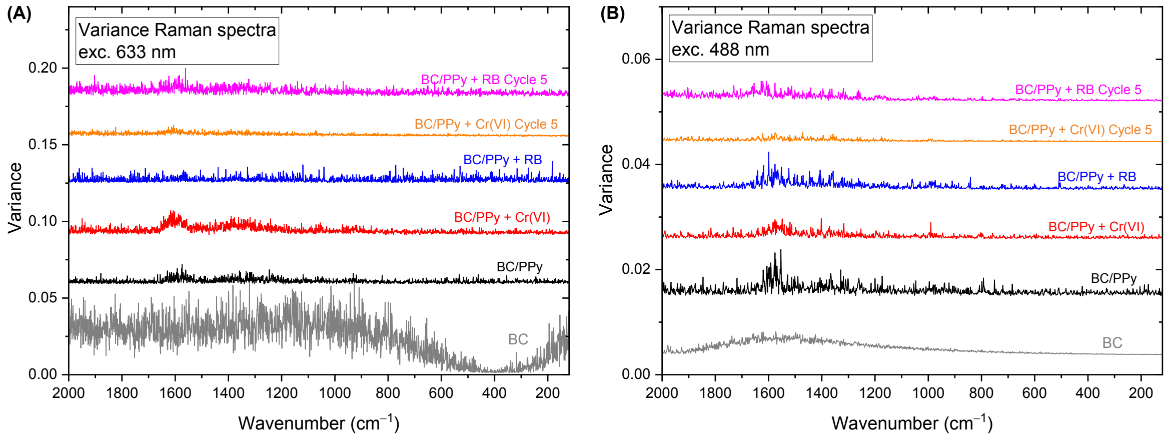

Fig. S4. Variance Raman spectra of BC, BC/PPy, samples with adsorbed RB and Cr(VI), and respective samples after 5 adsorption/desorption cycles, excited at 633 nm (A) and 488 nm (B). The spectra are shifted for clarity. The broadband in the BC spectra reflects variance in fluorescence shape. The scale of the variance spectra suggests very high homogeneity of all samples. This includes homogeneity of the PPy coating on BC, PPy molecular structure, and Cr(VI) ions and RB dye adsorption distribution.

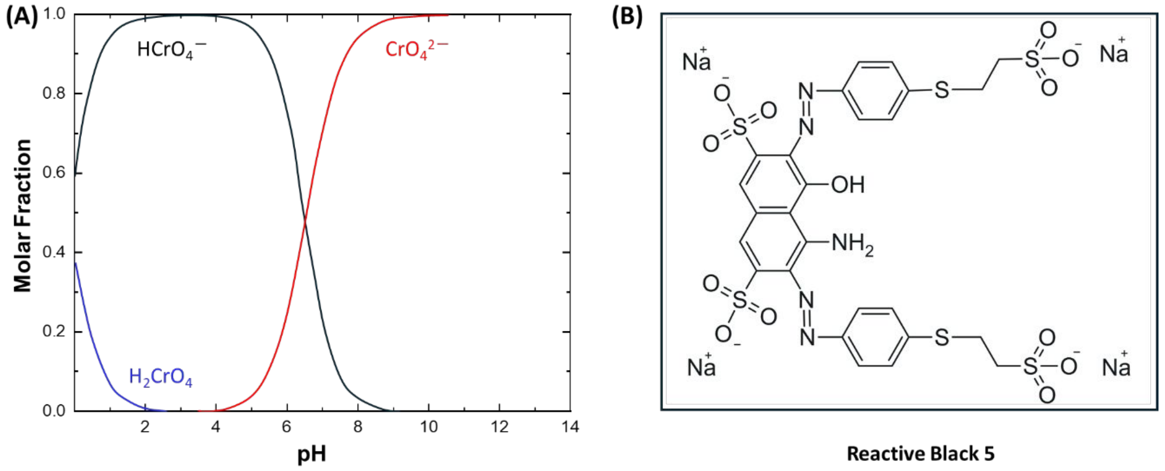

Fig. S5. Speciation of Cr(VI) ions at different pH levels (A), and the molecular structure of Reactive Black 5 (B).

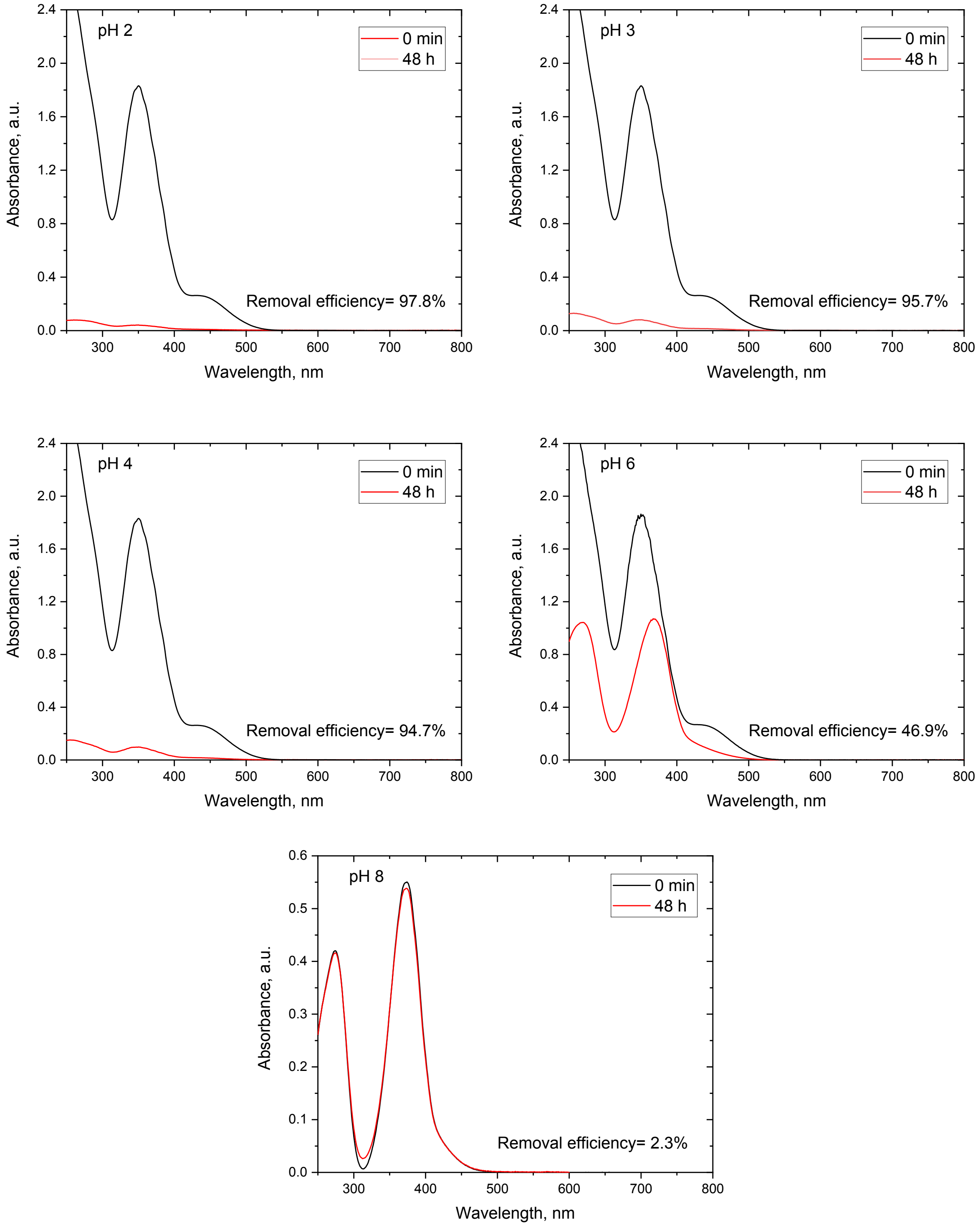

Fig. S6. Effect of pH on the removal efficiency of Cr(VI) (60 mg/L, 25 mL) using BC/PPy composite (5 mg). Spectra were recorded using a 1 cm path-length cell, except that at pH 8 were recorded with a 1 mm cell.

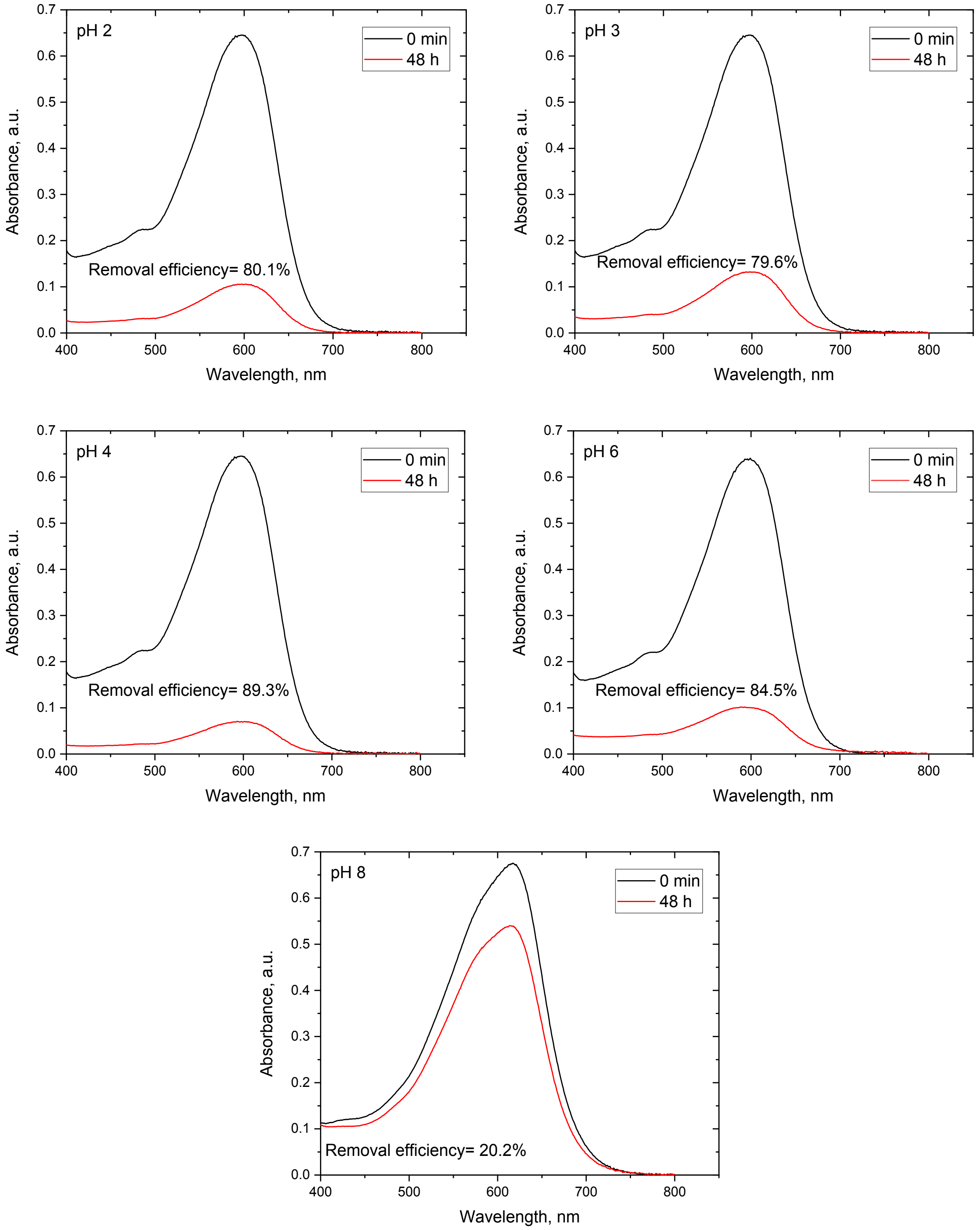

Fig. S7. Effect of pH on the removal efficiency of RB (20 mg/L, 25 mL) using BC/PPy (5 mg).

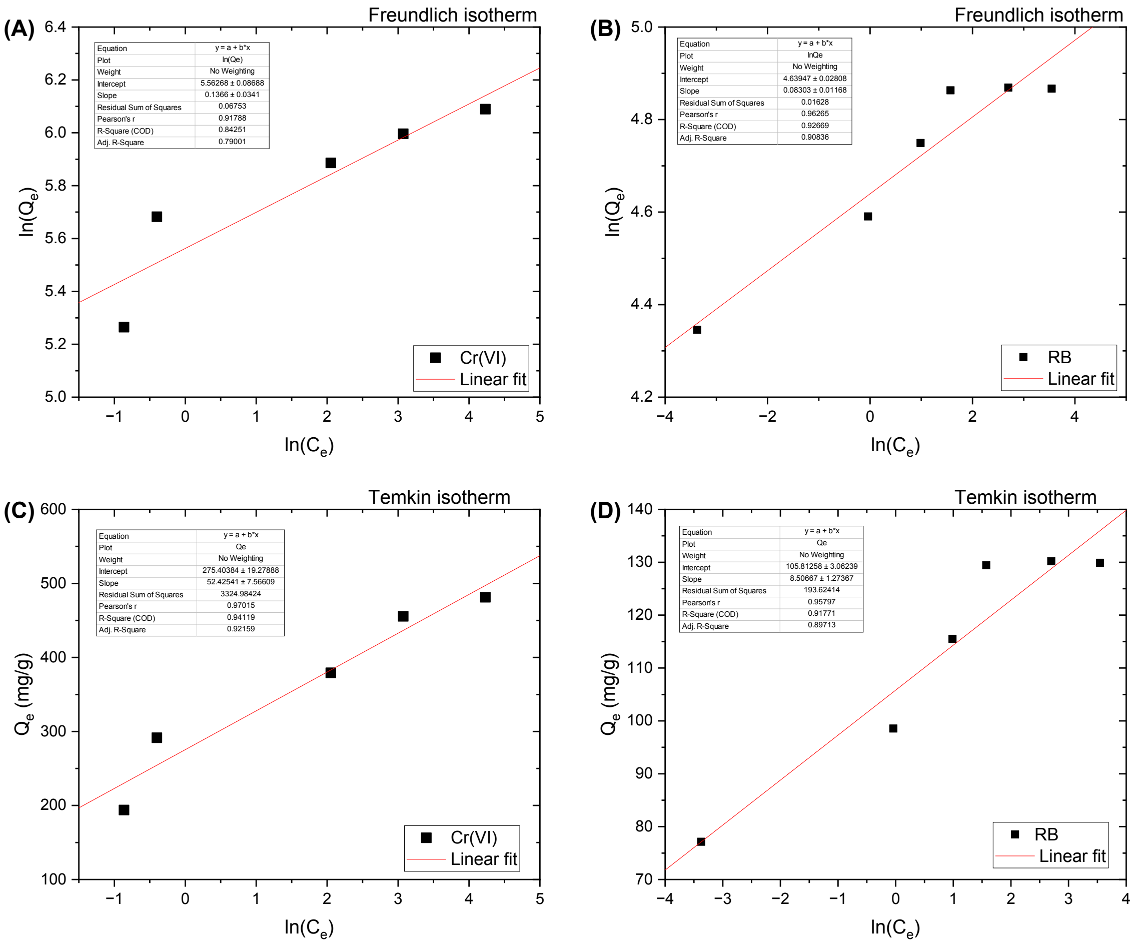

Fig. S8. Freundlich and Temkin isotherm modeling of Cr(VI) (A,C) and RB (B,D) adsorption onto BC/PPy.

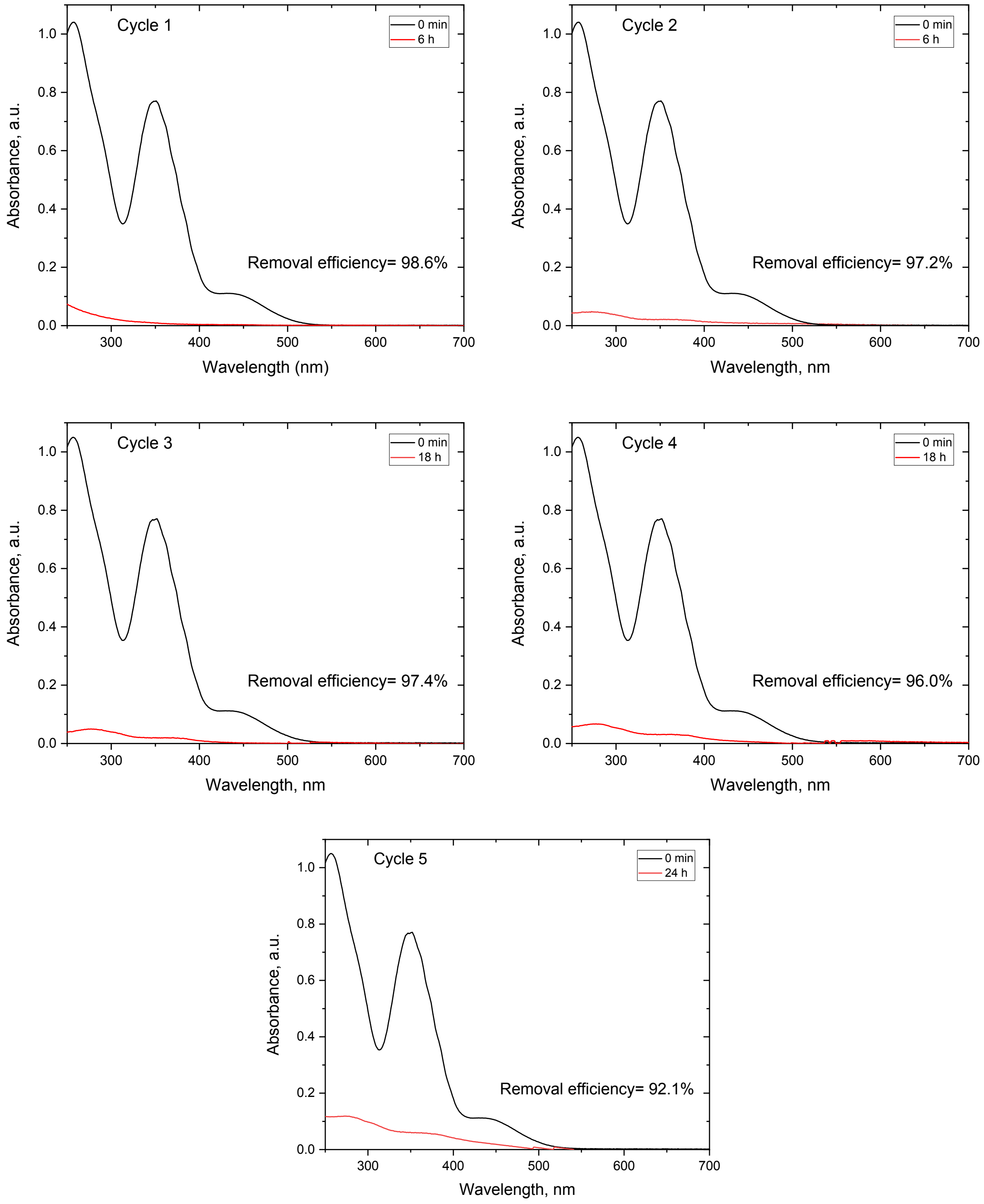

Fig. S9. Adsorption of Cr(VI) ions onto BC/PPy at different cycles.

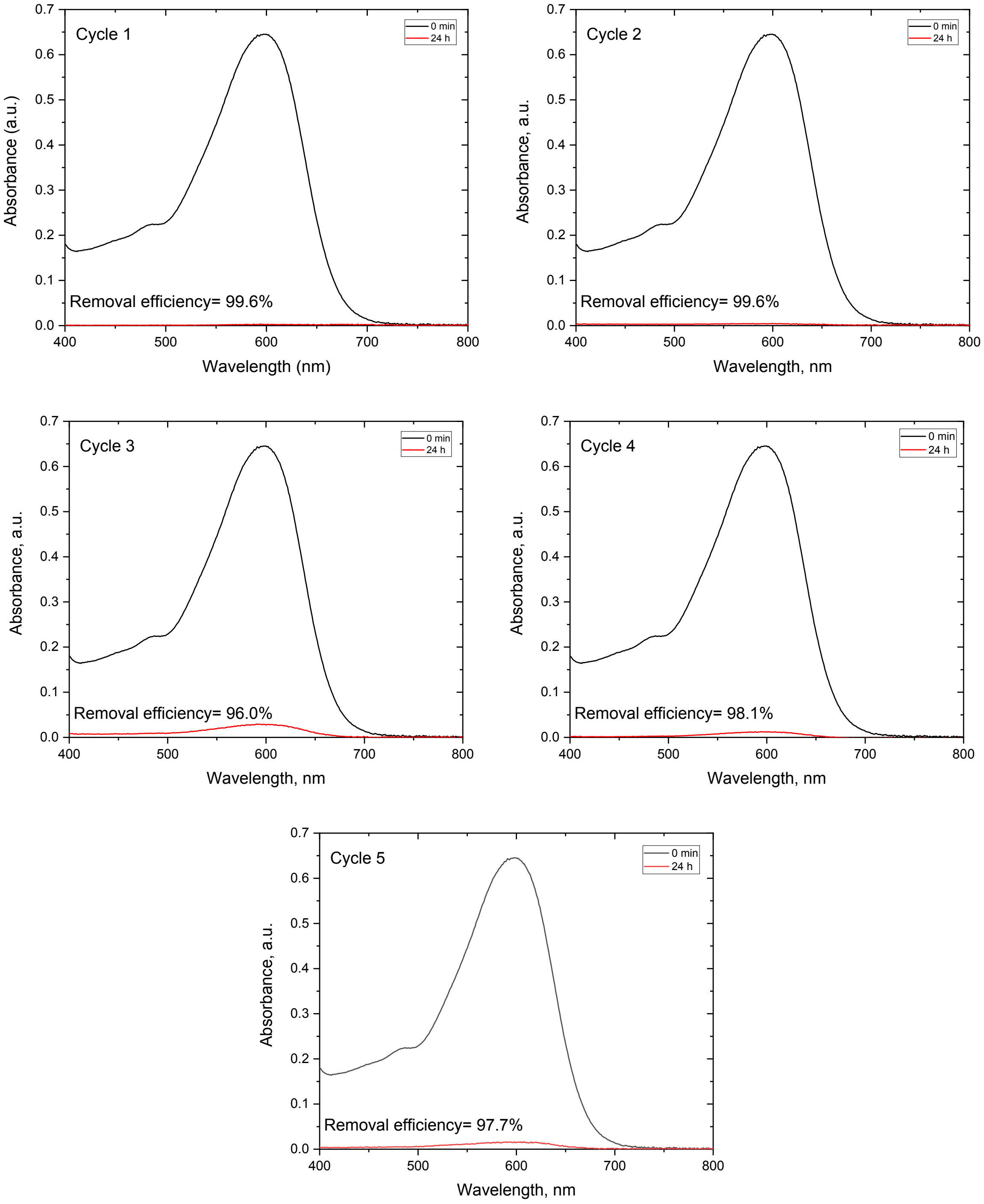

Fig. S10. Adsorption of RB onto BC/PPy at different cycles.

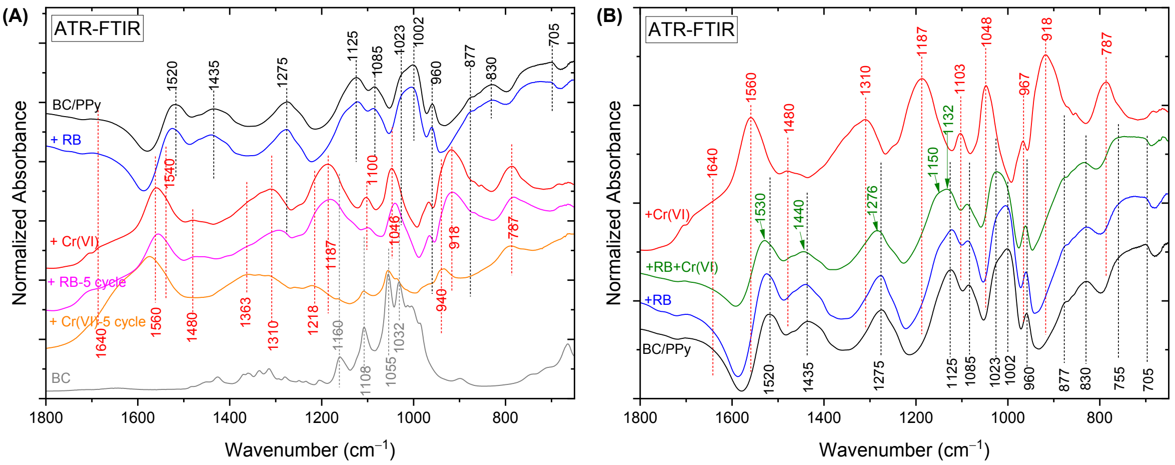

Fig. S11. FTIR spectra of BC, BC/PPy, samples with adsorbed RB and Cr(VI), and corresponding cycled samples (A), comparison of adsorption of RB, Cr(VI), and their binary mixture (B).

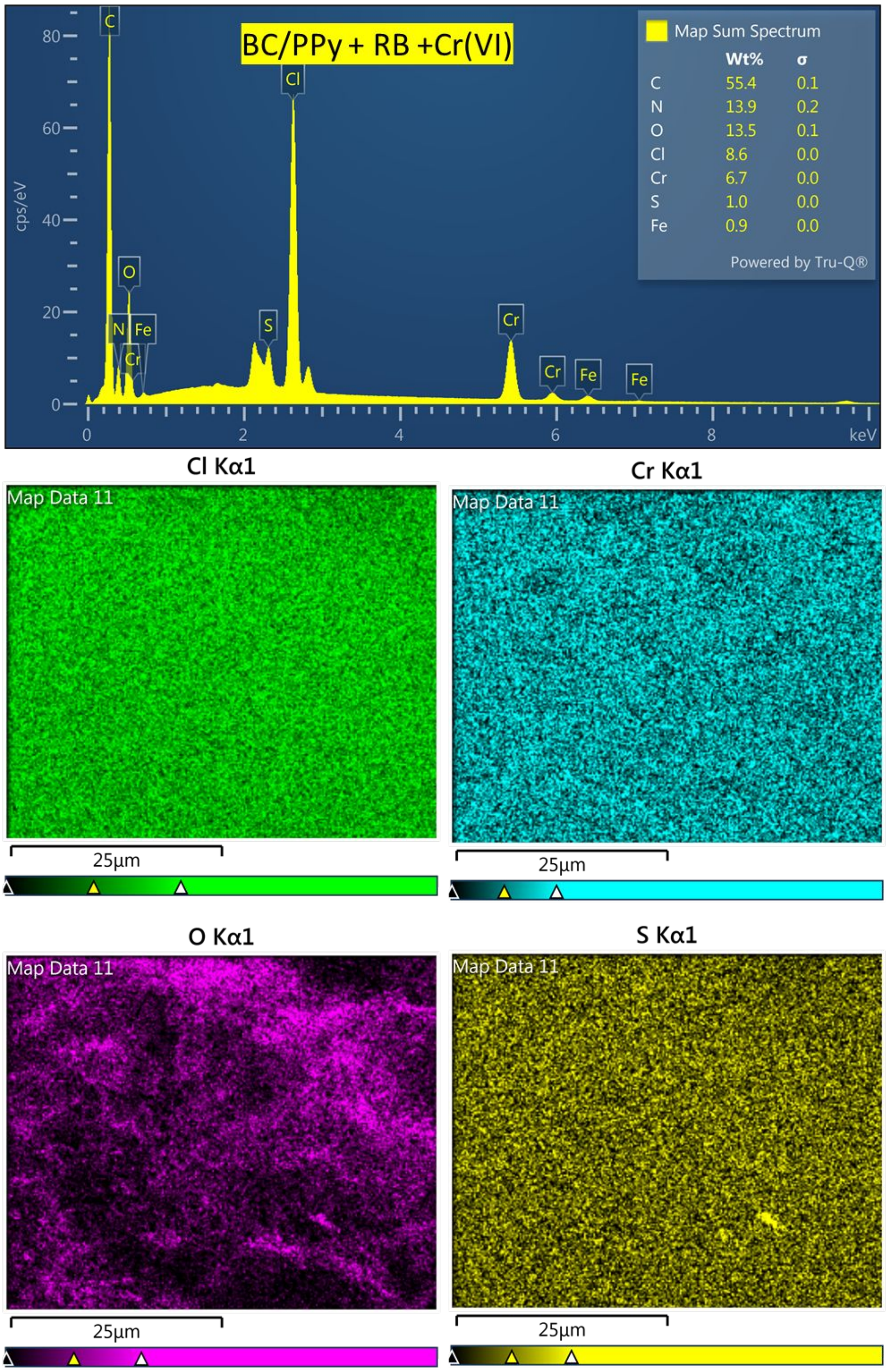

Fig. S12. EDX Spectra and elemental mapping of bacterial cellulose/polypyrrole composite after adsorption of Cr(VI) ions and Reactive Black 5, a binary mixture.
